# Carbasugar analogues of galactofuranosides: α-*O*-linked derivatives

**DOI:** 10.3762/bjoc.6.129

**Published:** 2010-11-29

**Authors:** Jens Frigell, Ian Cumpstey

**Affiliations:** 1 Department of Organic Chemistry, The Arrhenius Laboratory, Stockholm University, Stockholm 106 91, Sweden; 2 Institut de Chimie des Substances Naturelles, CNRS, 91198 Gif-sur-Yvette, France, Tel.: +33 (0)1 69 82 30 78; fax: +33 (0)1 69 07 72 47

**Keywords:** carbasugars, etherification, galactofuranose, glycomimetics, pseudodisaccharides, regioselective

## Abstract

Using an indirect method, we have synthesised α-linked carbasugar analogues of galactofuranosides for the first time. Ring opening of a β-*talo* configured carbasugar 1,2-epoxide by alcohol nucleophiles under Lewis acidic conditions proceeded with very good regioselectivity to give α-*talo* configured C1-substituted ethers with a free OH-group at the C2 position. Inversion of configuration at C2 by an oxidation–reduction sequence gave the α-*galacto* configured carbahexofuranose C1 ethers. A carbadisaccharide corresponding to the Gal*f*(α1→3)Man*p* substructure from *Apodus deciduus* galactomannan was synthesised to exemplify the method.

## Introduction

Galactofuranose is found in nature as a component of glycoconjugates in many microorganisms [1–2]. Galactofuranosides with the α configuration are rather less common than their β-linked anomers, from which they are distinguished by their ^13^C C1 chemical shifts (α, 103.8 ppm; β, 109.9 ppm for the methyl glycosides) [3], and ^3^*J*_1,2_^1^H,^1^H coupling constants (α, 4 Hz; β, 2 Hz) [4]. Nevertheless, α-galactofuranosides occur as components of a number of oligosaccharides, including plant polysaccharides [5], fungal cell wall polysaccharides [6], glycolipids from thermophilic bacteria [7] and glycosphingolipids from marine sponge (agelagalastatin) [8]. Moreover, the precursor to β-galactofuranosides and the substrate for galactofuranosyltransferases is UDP-galactofuranose, which has an α configuration [1–2]. Modified, hydrolytically stable analogues of galactofuranosides could find application in the design and synthesis of potential inhibitors of the enzymes involved in the biosynthesis of galactofuranose-containing glycoconjugates.

**Figure 1:**
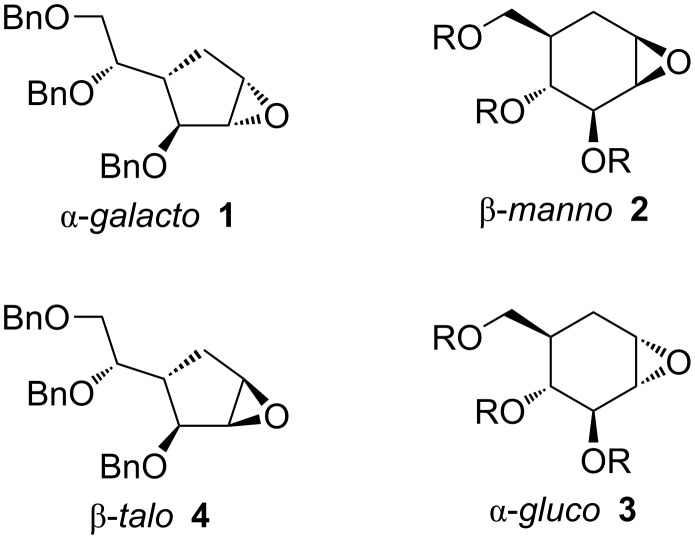
Structures of carbapyranose and carbafuranose 1,2-epoxides.

We have recently developed what appears to be a general route towards carbasugar analogues of β-galactofuranosides [9]. In our approach, alcohol nucleophiles attack an α-*galacto* carbasugar 1,2-epoxide **1** (Figure 1) under Lewis acidic conditions with excellent regioselectivity for attack at C1. The regioselectivity may be rationalised by steric and electronic arguments: C1 is more accessible than C2 due to the absence of a neighbouring bulky group, the C3 benzyl ether. C1 is more electrophilic than C2; it is better able to sustain partial positive charge under Lewis acid coordination due to the absence of a neighbouring electron-withdrawing group, the C3 benzyl ether.

Ogawa has extensively used epoxide-opening reactions as a coupling method to access *N*- and *O*-linked pseudodisaccharides based on carbapyranoses [10]. The epoxides were opened by amines in uncatalysed reactions, or by alcohols under Lewis acidic, but more usually basic, conditions. An important general feature of epoxide opening in 6-membered rings is the tendency for *trans*-diaxial ring opening [11]. The existing substituents on the cyclohexane ring favour one of the two possible half-chair conformations (^4^*H*_5_ for **1** and **2**). The principle of microscopic reversibility requires an antiperiplanar relationship between the nucleophile and the epoxide oxygen in the product immediately after reaction. From a given half-chair conformation of the epoxide, nucleophilic attack results in either a chair (with the substituents *trans*-diaxial) or a skew-boat conformation, which must rearrange to give the diequatorial product. As the skew-boat is a higher energy conformation, this situation is disfavoured from a stereoelectronic point of view.

Carbapyranose 1,2-epoxides with the β-*manno* configuration **2** (Figure 1) were opened efficiently with attack at C1 (sterically and electronically favoured) to give 1,2-*trans*-diaxial [11] carba-α-mannose derivatives with both oxygen [12] and nitrogen [13] nucleophiles. The electronic argument must be reformulated slightly in the absence of a Lewis acid. The localisation of electrons onto the leaving group (the epoxide oxygen) is necessary as the transition state is approached also under neutral or base-catalysed conditions [14]. Carbon C2 bears a β electron-withdrawing group (the C3 benzyl ether), whereas C1 does not. Hence, from an electronic point of view, S_N_2 reactions should be more favourable at C1 than at C2. The same concept is a major factor in explaining the generally low reactivity of carbohydrates (at non-anomeric positions) in S_N_2 reactions. Carbapyranose 1,2-epoxides with α-*gluco* **3** and α-*galacto* configurations did not give such good results. With amine nucleophiles, attack was often unregioselective [15]: attack at C1 (favoured sterically and electronically) leads to stereoelectronically unfavourable [11] 1,2-*trans*-diequatorial opening. This nucleophilic dilemma presumably accounts for the poor regioselectivity and yields of such reactions. With alcohol nucleophiles, no coupling products were observed with either α-*gluco* or α-*galacto* carbapyranose 1,2-epoxides [16].

We considered the reactivity of a diastereomeric epoxide to the α-*galacto* carbafuranose epoxide **1**, i.e., a β-*talo* epoxide **4**, in nucleophilic ring-opening reactions. The same steric and electronic arguments on regioselectivity would predict attack at C1 in the β-*talo* 1,2-epoxide **4**. In 5-membered rings, nucleophilic attack at either carbon of an epoxide (with an envelope conformation) would result in a product with a twist-boat conformation, meaning that there was no great difference in energy between the two regioisomers as was the case in the 6-membered ring series, irrespective of the preferred envelope conformation of the epoxide, and that stereoelectronic effects are thus likely to be much less relevant for the more flexible cyclopentane derivatives. One factor that could speak against good regioselectivity in this case would be the fact that the α-*talo* product has a 2,3-*cis* and 1,4-*cis* substituent pattern, which could lead to some strain in the transition state, whereas β-*galacto* derivatives have an all *trans* substituent pattern.

Should the epoxide-opening reaction proceed to give the α-*talo* configured C1-substituted products, then this could offer a route to α-configured galactofuranoside pseudodisaccharides via a subsequent epimerisation of the unprotected C2 alcohol, analogous to the α-*manno* to α-*gluco* epimerisation route developed by Ogawa in the six-membered ring series [17]. In this paper we report our results on the synthesis of carbasugar analogues of α-galactofuranosides by this route.

Some related work in the form of ring opening of carbapentafuranose 1,2-epoxides has been reported in the area of carbanucleoside chemistry. Indeed, various α-*ribo* configured carbasugar epoxides, which have the same configuration around the carbocyclic ring as the β-*talo* epoxide **4**, were opened by nitrogen nucleophiles (nucleobases [18] or azide [19–20]) with generally good regioselectivity for attack at C1. The only precedent for attack at such carbapentafuranose epoxides with an oxygen nucleophile would appear to be the acid-mediated attack of water on an α-*xylo* configured epoxide, again with apparently good regioselectivity for attack at C1 [21].

## Results and Discussion

**Scheme 1:**
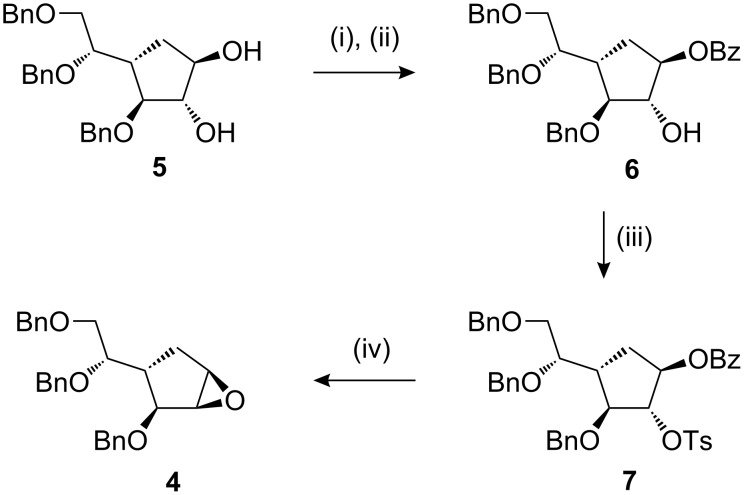
(i) Bu_2_SnO, MeOH, 70 °C; (ii) BzCl, toluene, 82%; (iii) TsCl, py, 50 °C, 86%; (iv) NaOMe, MeOH, 81%.

Our synthesis of the β-*talo* 1,2-epoxide began from the β-*galacto* 1,2-diol **5** [22]. We needed to regioselectively transform OH-2 into a leaving group that could then undergo intramolecular displacement by O-1. The OH-1 hydroxyl group appears to be more nucleophilic than OH-2 in **5** [9]; a benzoate protecting group was introduced at O-1 using a tin acetal method [23] to give the monobenzoate **6** with enhanced regioselectivity (Scheme 1). Only very small amounts (<5%) of the regioisomer were observed and were not isolated in a pure form. The free OH-2 could then be converted to its tosylate **7**. Treatment of this diester **7** with sodium methoxide resulted in the direct formation of the required epoxide **4**, presumably by cleavage of the C1 benzoate to the alcoholate (with retention of configuration) followed by intramolecular displacement of the C2 tosylate (with inversion of configuration). We synthesised the same epoxide **4** (78%) by treatment of isolated 3,5,6-tri-*O*-benzyl-2-*O*-(toluene-4-sulfonyl)-4a-carba-β-D-galactofuranose [9] with sodium hydride in DMF.

**Scheme 2:**
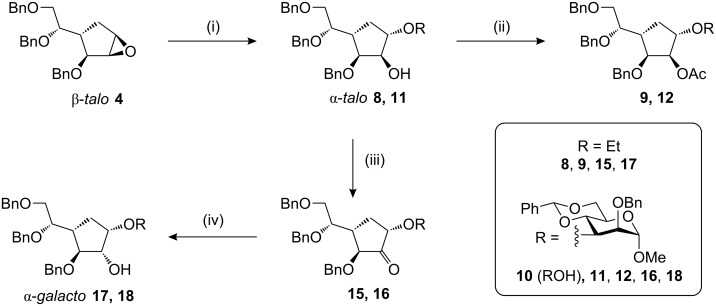
(i) ROH, BF_3_^.^OEt_2_, CH_2_Cl_2_, RT; **8**, 90%; **11**, 57%; (ii) Ac_2_O, py; **9**, >99%; **12**, 91%; (iii) (COCl)_2_, DMSO, CH_2_Cl_2_, −60 °C, then Et_3_N, RT; **15**, 88%; **16**, 68%; (iv) L-selectride, THF, 0 °C; **17**, 78%; **18**, 66%.

We investigated ring-opening of the epoxide **4** by alcohol nucleophiles under Lewis acidic conditions as used in the diastereomeric series [9]. With excess ethanol (10 equiv), we obtained the C1 ethyl ether **8** as the major product (90%) along with a small amount (4%) of the regioisomer (Scheme 2). The regiochemistry was proved by acetylation of the free alcohols. The major product **8** gave a C2 acetate **9**, as shown by the downfield shift of H2 in the ^1^H NMR spectrum, indicating nucleophilic attack at C1 during the epoxide-opening step to give an α-*talo* configured product. The minor product gave a C1 acetate on acetylation, indicating attack at C2 in the epoxide opening and formation of a β-*galacto* product.

**Figure 2:**
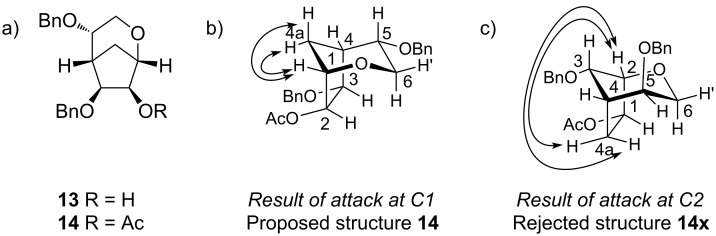
a) Structures of **13** and **14**; b) observed NOEs consistent with structure **14**; c) observed NOEs inconsistent with structure **14x**.

We then examined a secondary carbohydrate alcohol as the nucleophile. The mannose OH-3 nucleophile **10** [24] would lead to a structure corresponding to a Gal*f*(α1→3)Man*p* disaccharide substructure from the galactomannan from the fungus *Apodus deciduus* [6]. Here also, the epoxide **4** underwent ring opening with very good regioselectivity in favour of the C1-substituted (α-*talo*) product **11**, which was isolated in 57% yield and characterised as its C2 acetate **12**. The regioisomeric C2-substituted (β-*galacto*) product was not detected in the epoxide-opening reaction. In this reaction, a by-product **13** was also formed, which was characterised as its acetate **14** and assigned the structure given in Figure 2a.

The ^1^H NMR spectrum of the by-product **13** showed the presence of only two sets of characteristic signals for benzyl ethers, indicating that the third benzyl ether protecting group had been lost. The mass spectrum showed a peak at *m*/*z* 363, consistent with a bicyclic system. Acetylation with acetic anhydride and pyridine gave a monoacetate **14** according to NMR spectroscopy and mass spectrometry (*m*/*z* 405).

We deduced the structure of the monoacetate **14** using NMR spectroscopy, including HSQC, HMBC and NOESY experiments. The C1/H1, C2/H2 and C3/H3 pairings could not be easily distinguished due to the presence of some long-range couplings and correlations in the COSY spectra. One of these three carbons bore an acetate, as seen from the downfield shift of the corresponding proton. The second bore a benzyl ether, as shown by the PhC*H*_2_→C cross-peaks in the HMBC spectrum. The third was linked to C6 by an ether, as seen from the H6’→C cross-peak in the HMBC spectrum. The HMBC spectrum also revealed that O-5 was still benzyl-ether-protected; hence, the benzyl ether was lost from O-6, presumably following nucleophilic attack by O-6 on either C1 or C2 of the epoxide. The large difference in chemical shift between H6 (3.15 ppm) and H6’ (3.90 ppm) is consistent with C6 being part of a rigid cyclic structure, rather than exocyclic.

The answer to the question of whether attack occurred at C1 or C2 required information from NOESY experiments. Attack at C1 would have led to **14**, whereas attack at C2 would have led to **14x**. The strongest support for the proposed structure was the NOE observed (Figure 2b) between H1 (if attack occurred at C1) and H4a and H4a’, which is consistent with structure **14**; this would correspond to an NOE (Figure 2c) between H2 (if attack occurred at C2) and H4a and H4a’, which is not consistent with structure **14x**. No NOE was seen between the acetylated H (H1 or H2) and H4a or H4a’: This supports structure **14** and essentially excludes the alternative structure **14x**. Finally, an NOE was observed between H6 (but not H6’) and the acetylated H (H1 or H2), which allowed the C6 protons to be distinguished. H6 appeared as an apparent triplet with *J* 11 Hz in the ^1^H NMR spectrum, which is consistent with a diaxial coupling to H5 as seen in structure **14**, but not in **14x**.

Hence, compound **13** presumably arose from debenzylative cycloetherification by attack of O-6 on C1, C1 being the most electrophilic carbon in both inter- and intramolecular epoxide-opening reactions.

To achieve the inversion of stereochemistry at C2 of the α-*talo* carbasugars, our best results were obtained using an oxidation–reduction approach. Attempted S_N_2 reactions (Mitsunobu reaction or sulfonate displacement) gave inferior results. Hence the C2 alcohols **8** and **11** were oxidised under Swern conditions to give the ketones **15** and **16**. Reduction of ketone group in ethyl ether **15** with sodium borohydride gave a rather poor diastereoselectivity. However, using L-selectride excellent selectivity for the formation of the required α-*galacto* carbasugar **17** (78% isolated) was observed; the α-*talo* product **8** was also formed, but not in sufficient quantity that it could be isolated in a pure state. The pseudodisaccharide ketone **16** was also then reduced with L-selectride, again very good diastereoselectivity for the α-*galacto* configured carbasugar **18** was observed.

## Conclusion

Complementing our approach to β-configured *O*-linked galactofuranosides by epoxide ring opening, the corresponding α-compounds can be made by an indirect route: Opening of a β-*talo* configured epoxide followed by C2 epimerisation. The very good regioselectivity in the epoxide-opening reaction indicates that steric and/or electronic effects favouring attack at C1 over C2 are more important than other factors; the difference in efficiency of the “up” (β-*manno*) and “down” (α-*gluco*) carbapyranose 1,2-epoxides in coupling reactions was not observed in the carbafuranose series. It is possible that this strategy for pseudodisaccharide formation by epoxide ring opening in the (α and β) carbagalactofuranose series could be extended to the pseudoenantiomeric (β and α) carbaarabinofuranose series [25], which represent similarly relevant biological targets [26].

## Supporting Information

Supporting information features experimental section and NMR spectra for new compounds.

File 1 Experimental and NMR data.

File 2 NMR spectra.
