# Supplementary material for: Carbasugar analogues of galactofuranosides: α-O-linked derivatives
Source: Beilstein J Org Chem. 2010 Nov 29;6:1127–31. doi: 10.3762/bjoc.6.129 (PMC3001987; doi:10.3762/bjoc.6.129)
Supplement: File 1 — Experimental and NMR data. [file Beilstein_J_Org_Chem-06-1127-s001.pdf]

Supporting Information

for

Carbasugar analogues of galactofuranosides: α-*O*-linked derivatives

Jens Frigell[1] and Ian Cumpstey[*1,2]

Address: [1]Department of Organic Chemistry, The Arrhenius Laboratory, Stockholm University, Stockholm 106 91, Sweden and [2]Institut de Chimie des Substances Naturelles, CNRS, 91198 Gif-sur-Yvette, France, Tel.: +33 (0)1 69 82 30 78; fax: +33 (0)1 69 07 72 47

Email: Ian Cumpstey - ian.cumpstey@icsn.cnrs-gif.fr; ian.cumpstey@sjc.oxon.org

* Corresponding author

Contents

Experimental section and NMR data for new compounds

Experimental section

General methods

Melting points were measured with a Büchi 510 melting point apparatus and are uncorrected. Proton nuclear magnetic resonance (^{1}H) spectra were recorded on Bruker Avance II 500 (500 MHz) or Bruker Avance II 400 (400 MHz) spectrometers; multiplicities are quoted as singlet (s), doublet (d), doublet of doublets (dd), doublet of doublet of doublets (ddd), triplet (t), apparent triplet (at), doublet of apparent triplets (dat), doublet of doublet of apparent triplets (ddat), quartet (q), apparent quartet (aq), apparent quartet of doublets (aqd) and multiplet (m). Carbon nuclear magnetic resonance (^{13}C) spectra were recorded on Bruker Avance II 500 (125 MHz) or Bruker Avance II 400 (100 MHz) spectrometers. ^{1}H and ^{13}C spectra and ^{13}C multiplicities were assigned using

COSY, HSQC and DEPT experiments. All chemical shifts are quoted on the δ-scale in parts per million (ppm). Residual solvent signals or TMS were used as an internal reference. Low- and high-resolution (HRMS) electrospray (ESI) mass spectra were recorded using a Bruker Microtof instrument. Infrared spectra were recorded on a Perkin-Elmer Spectrum One FT-IR spectrometer using the thin film method on NaCl plates. Optical rotations were measured on a Perkin-Elmer 241 polarimeter with a path length of 1 dm; concentrations are given in g/100 mL. Thin layer chromatography (TLC) was carried out on Merck Kieselgel sheets, pre-coated with $60F_{254}$ silica. Plates were visualised with UV light and developed using 10% sulfuric acid, or an ammonium molybdate (10% w/v) and cerium (IV) sulfate (2% w/v) solution in 10% sulfuric acid. Flash column chromatography was carried out on silica gel (35–70 µm, Grace). Dichloromethane was distilled from calcium hydride. Diethyl ether, THF and toluene were dried and dispensed from Vacuum Atmospheres solvent purification columns. Reactions performed under an atmosphere of nitrogen were maintained by an inflated balloon.

1-*O*-Benzoyl-3,5,6-tri-*O*-benzyl-4a-carba-β-D-galactofuranose (6)

Diol **5** (208 mg, 0.464 mmol) was dissolved in MeOH (5 mL) at RT. Bu_2SnO (254 mg, 1.02 mmol) was added, the mixture refluxed for 2 h and then all solvent was removed *in vacuo*. The crude mixture was dissolved in toluene (5 mL), and BzCl (102 µL, 0.082 mmol) was added slowly at 0 °C. The reaction was stirred at RT for 18 h after which TLC (toluene–EtOAc, 4:1) showed complete consumption of starting material (R_f 0) and formation of a single product (R_f 0.7). The reaction was quenched by the addition of KF (satd. aq., 5 mL). The mixture was washed with KF (satd. aq., 3 × 5 mL), and the aqueous phase extracted with EtOAc (2 × 10 mL). The combined organic phases were dried ($MgSO_4$), filtered and concentrated *in vacuo*. The crude product was purified by column chromatography (pentane–EtOAc, 6:1) to give alcohol **6** (210 mg, 82%) as an oil; $[\alpha]_D^{25}$ −38.0 (c, 1.0 in $CHCl_3$); δ_H (400 MHz, $CDCl_3$) 1.96 (1H, ddd, $J_{1,4a}$ 2.8 Hz, $J_{4,4a}$ 8.4 Hz, $J_{4a,4a'}$ 14.0 Hz, H-4a), 2.30 (1H, ddd, $J_{1,4a'}$ 8.1 Hz, $J_{4,4a'}$ 10.4 Hz, $J_{4a,4a'}$ 14.3 Hz, H-4a'), 2.47 (1H, m,

H-4), 3.57 (1H, dd, $J_{5,6}$ 4.9 Hz, $J_{6,6'}$ 10.0 Hz, H-6), 3.63 (1H, dd, $J_{5,6'}$ 6.1 Hz, $J_{6,6'}$ 10.0 Hz, H-6'), 3.82–3.87 (2H, m, H-3, H-5), 4.22 (1H, m, H-2), 4.42, 4.74 (2H, 2 × d, *J* 11.5 Hz, PhC*H*$_2$), 4.50, 4.76 (2H, 2 × d, *J* 11.6 Hz, PhC*H*$_2$), 4.52, 4.58 (2H, 2 × d, *J* 12.1 Hz, PhC*H*$_2$), 4.97 (1H, dat, J_{at} 3.5 Hz, $J_{1,4a'}$ 8.1 Hz, H-1), 7.26–7.36 (15H, m, Ar-H), 7.42–7.58 (3H, m, Ar-H), 8.02–8.05 (2H, m, Ar-H); δ_C (125 MHz, $CDCl_3$) 27.3 (t, C-4a), 43.9 (d, C-4), 72.2, 72.3, 73.1, 73.5 (4 × t, C-6, 3 × Ph*C*H$_2$), 76.4, 84.8 (2 × d, C-3, C-5), 81.9 (d, C-1), 83.7 (d, C-2), 127.7, 127.8, 127.8, 127.9, 128.1, 128.2, 128.2, 128.5, 128.5, 128.5, 128.5, 129.9 (12 × d, Ar-*C*H), 133.4 (s, Ar-*C*C=O), 138.3, 138.6, 138.7 (3 × s, Ar-C), 167.9 (s, C=O); IR 1717 (C=O), 3448 (OH) cm^{-1}; HRMS Calcd. For $C_{35}H_{36}O_6Na$ (MNa^+) 575.2404. Found 575.2399.

1-*O*-Benzoyl-3,5,6-tri-*O*-benzyl-2-*O*-(toluene-4-sulfonyl)-4a-carba-β-D-galactofuranose (7)

Alcohol **6** (210 mg, 0.380 mmol) was dissolved in pyridine (5 mL) at RT and TsCl (435 mg, 2.28 mmol) added. After 18 h, TLC (toluene–EtOAc, 10:1) showed consumption of starting material (R_f 0.3) and formation of a product (R_f 0.7). HCl (1 M, 5 mL) was added to the crude reaction mixture, which was then extracted with EtOAc (3 × 10 mL). The combined organic phases were dried ($MgSO_4$), filtered and concentrated *in vacuo*. The crude product was purified by column chromatography (toluene–EtOAc, 12:1) to give tosylate **7** (231 mg, 86%) as white crystals, m.p. 87–89 °C (EtOAc–Et_2O); $[\alpha]_D^{25}$ −71.3 (c, 1.0 in $CHCl_3$); δ_H (400 MHz, $CDCl_3$) 1.70 (1H, ddd, *J* 2.3 Hz, *J* 8.0 Hz, $J_{4a,4a'}$ 14.1 Hz, H-4a), 2.20–2.28 (4H, m, H-4a', ArC*H*$_3$), 2.41 (1H, m, H-4), 3.43 (1H, dd, $J_{5,6}$ 5.2 Hz, $J_{6,6'}$ 10.0 Hz, H-6), 3.54 (1H, dd, $J_{5,6'}$ 5.9 Hz, $J_{6,6'}$ 9.9 Hz, H-6'), 3.74 (1H, m, H-5), 4.03 (1H, dd, *J* 5.5 Hz, *J* 8.8 Hz, H-3), 4.32 (1H, d, *J* 11.6 Hz, PhC*H*H'), 4.33 (1H, d, *J* 11.4 Hz, PhC*H*H'), 4.43, 4.50 (2H, 2 × d, *J* 12.1 Hz, PhC*H*$_2$), 4.66–4.69 (2H, m, 2 × PhCH*H*'), 5.09 (1H, dat, J_{at} 3.3 Hz, *J* 7.2 Hz, H-1), 5.13 (1H, m, H-2), 7.10 (2H, m, Ar-H), 7.24–7.62 (18H, m, Ar-H), 7.77 (2H, m, Ar-H), 7.94 (2H, m, Ar-H); δ_C (100 MHz, $CDCl_3$) 21.5 (q, Ar*C*H$_3$), 28.4 (t, C-4a), 43.6 (d, C-4), 71.6 (t, C-6), 72.5, 72.7, 73.4 (3 × t, 3 × Ph*C*H$_2$), 75.5 (d, C-5), 76.4 (d, C-1), 82.8 (d, C-3), 89.6 (d, C-2), 127.6, 127.7, 127.9, 127.9, 128.3, 128.4, 128.4, 129.7, 129.8, 129.9 (10 × d, Ar-

CH), 133.3, 133.6 (2 × s, Ar-*C*SO_2, Ar-*C*C=O), 137.8, 138.1, 138.5 (3 × s, 3 × Ar-C), 145.1 (s, Ar-*C*CH_3), 165.0 (s, C=O); IR 1721 (C=O) cm^{-1}; HRMS Calcd. For $C_{42}H_{42}O_8NaS$ (MNa^+) 729.2472. Found 729.2493.

1,2-Anhydro-3,5,6-tri-*O*-benzyl-4a-carba-β-D-talofuranose (4)

Tosylate **7** (220 mg, 0.311 mmol) was suspended in MeOH (5 mL). Na (20 mg, 0.85 mmol) was added to MeOH (5 mL) and the resulting solution of NaOMe was added to the reaction vessel at RT. The reaction mixture was stirred for 18 h. TLC (toluene–EtOAc, 10:1) showed steady formation of a single product (R_f 0.4) and consumption of starting material (R_f 0.7). After 18 h, the reaction was complete and it was quenched by the addition of $NH_4Cl_{(s)}$ (90 mg, 1.7 mmol). All the MeOH was removed *in vacuo*, the crude product dissolved in EtOAc (30 mL), and washed with H_2O (2 × 30 mL). The combined aqueous phases were extracted with EtOAc (2 × 30 mL). The combined organic extracts were dried ($MgSO_4$), filtered and concentrated *in vacuo*. The crude product was purified by column chromatography (toluene–EtOAc, 12:1) to give epoxide **4** (108 mg, 81%) as an oil; $[\alpha]_D^{25}$ −84.9 (c, 1.0 in $CHCl_3$); δ_H (400 MHz, $CDCl_3$) 1.76 (1H, m, H-4a), 1.91–2.03 (2H, m, H-4a', H-4), 3.41 (1H, d, *J* 2.6 Hz, H-1), 3.46–3.50 (2H, m, $J_{5,6}$ 4.5 Hz, H-2, H-6), 3.56 (1H, dd, $J_{5,6'}$ 6.4 Hz, $J_{6,6'}$ 10.0 Hz, H-6'), 3.76 (1H, m, H-5), 3.90 (1H, dd, $J_{2,3}$ 1.0 Hz, $J_{3,4}$ 7.6 Hz, H-3), 4.31, 4.71 (2H, 2 × d, *J* 11.6 Hz, PhCH_2), 4.45, 4.65 (2H, 2 × d, *J* 12.0 Hz, PhCH_2), 4.49, 4.53 (2H, 2 × d, *J* 12.1 Hz, PhCH_2), 7.22–7.35 (15H, m, Ar-H); δ_C (100 MHz, $CDCl_3$) 25.9 (t, C-4a), 40.0 (d, C-4), 54.9 (d, C-1), 55.2 (d, C-2), 71.7, 72.8, 72.9, 73.5 (4 × t, 3 × Ph*C*H_2, C-6), 76.0 (d, C-5), 80.8 (d, C-3), 127.6, 127.7, 127.7, 127.9, 128.0, 128.1, 128.4, 128.5, 128.5 (9 × d, Ar-CH), 138.3, 138.5, 138.9 (3 × s, Ar-C); HRMS Calcd. For $C_{28}H_{30}O_4Na$ (MNa^+) 453.2036. Found 453.2018.

3,5,6-Tri-*O*-benzyl-1-*O*-ethyl-4a-carba-α-D-talofuranose (8)

Epoxide **4** (51 mg, 0.119 mmol) was dissolved in CH_2Cl_2 (0.75 mL) at RT under a N_2 atmosphere and EtOH (63 µL, 1.19 mmol) was added. BF_3Et_2O (15 µL, 0.12 mmol) was dissolved in dry CH_2Cl_2 (1.5 mL), and 150 µL (12 µmol BF_3Et_2O) of this solution transferred to the colourless reaction mixture, which instantly turned pale yellow. After 24 h, TLC (toluene–EtOAc, 6:1) showed formation of a major product (R_f 0.3) and consumption of epoxide (R_f 0.6). The reaction was quenched by the addition of Et_3N (0.1 mL) and concentrated *in vacuo*. The crude product was purified by column chromatography (toluene–EtOAc, 10:1) to yield ethyl ether **8** (51 mg, 90%) as an oil; $[\alpha]_D^{25}$ −30.5 (c, 1.0 in $CHCl_3$); δ_H (400 MHz, $CDCl_3$) 1.19 (3H, t, *J* 7.0 Hz, CH_2CH_3), 1.56 (1H, ddd, $J_{4a,4a'}$ 13.3 Hz, *J* 9.9 Hz, *J* 6.5 Hz, H-4a), 2.06 (1H, ddd, $J_{4a,4a'}$ 13.3 Hz, *J* 7.3 Hz, *J* 8.6 Hz, H-4a’), 2.23 (1H, m, H-4), 3.50–3.61 (4H, m, H-6, H-6’, CH_2CH_3), 3.69–3.76 (2H, m, H-1, H-5), 3.81 (1H, at, *J* 6.4 Hz, H-3), 3.96 (1H, m, H-2), 4.36, 4.46 (2H, 2 × d, *J* 11.4 Hz, $PhCH_2$), 4.42, 4.74 (2H, 2 × d, *J* 11.8 Hz, $PhCH_2$), 4.49, 4.55 (2H, 2 × d, *J* 12.1 Hz, $PhCH_2$), 7.26–7.35 (15H, m, Ar-H); δ_C (100 MHz, $CDCl_3$) 15.5 (q, CH_2CH_3), 27.6 (t, C-4a), 43.1 (d, C-4), 65.1 (t, CH_2CH_3), 72.3, 72.5, 72.8, 73.6 (4 × t, 3 × $PhCH_2$, C-6), 74.5 (d, C-2), 77.3, 83.4 (2 × d, C-1, C-5), 80.5 (d, C-3), 127.7, 127.8, 127.8, 127.8, 128.0, 128.2, 128.4, 128.5, 128.7 (9 × d, Ar-CH), 137.8, 138.3, 138.8 (3 × s, Ar-C); IR 3431 (OH) cm^{-1}; HRMS Calcd. For $C_{30}H_{36}NaO_5$ (MNa^+) 499.2455. Found 499.2473.

Traces of the regioisomeric β-*galacto* 2-*O*-ethyl ether (2 mg, 4%) were isolated as an oil; δ_H (500 MHz, $CDCl_3$) 1.22 (3H, t, *J* 7.0 Hz, CH_2CH_3), 1.68 (1H, ddd, $J_{1,4a}$ 4.0 Hz, $J_{4,4a}$ 8.7 Hz, $J_{4a,4a'}$ 13.7 Hz, H-4a), 2.01 (1H, ddd, $J_{1,4a'}$ 6.8 Hz, $J_{4,4a'}$ 9.2 Hz, $J_{4a,4a'}$ 13.7 Hz, H-4a’), 2.37 (1H, m, H-4), 3.50–3.57 (7H, m, H-2, H-3, H-5, H-6, H-6’, CH_2CH_3), 4.06 (1H, dat, J_{at} 4.2 Hz, $J_{1,4a'}$ 6.7 Hz, H-1), 4.96, 5.21 (2H, 2 × d, *J* 11.7 Hz, $PhCH_2$), 5.00, 5.13 (2H, 2 × d, *J* 11.7 Hz, $PhCH_2$), 5.03, 5.07 (2H, 2 × d, *J* 12.0 Hz, $PhCH_2$), 7.25–7.34 (15H, m, Ar-H); δ_C (100 MHz, $CDCl_3$) 15.8 (q, CH_2CH_3), 31.5 (t, C-4a), 43.5 (d, C-4), 65.7 (t, CH_2CH_3), 72.1, 72.4, 73.0, 73.5 (4 × t, 3 × $PhCH_2$, C-6), 74.9 (d, C-1), 77.3, 80.5, 83.4 (3 × d, C-2, C-3, C-5), 127.6, 127.7, 128.0, 128.1, 128.4, 128.5, 128.5 (7 × d, Ar-

CH), 138.4, 138.6, 139.0 (3 × s, Ar-C); HRMS Calcd. For $C_{30}H_{36}NaO_5$ (MNa^+) 499.2455. Found 499.2453.

The α-*talo* 1-*O*-ethyl ether **8** (9 mg, 0.02 mmol) was dissolved in Ac_2O–pyridine, 1:1 (1 mL) and stirred at RT for 1 h, after which time TLC (toluene–EtOAc, 6:1) showed formation of a single product (R_f 0.8) and no remaining starting material (R_f 0.3). All the solvent was removed *in vacuo* and the crude product purified by column chromatography (toluene–EtOAc, 8:1) to give acetate **9** (12 mg, quant.) as an oil; δ_H (400 MHz, $CDCl_3$) 1.17 (3H, t, *J* 7.0 Hz, CH_2CH_3), 1.64 (1H, ddd, $J_{1,4a}$ 5.8 Hz, $J_{4,4a}$ 9.9 Hz, $J_{4a,4a'}$ 13.5 Hz, H-4a), 2.07 (3H, s, C(O)CH_3), 2.08 (1H, ddd, $J_{1,4a'}$ 7.5 Hz, $J_{4,4a'}$ 8.6 Hz, $J_{4a,4a'}$ 13.4 Hz, H-4a'), 2.24 (1H, aqd, J_{aq} 8.8 Hz, $J_{4,5}$ 3.7 Hz, H-4), 3.45–3.59 (4H, m, H-6, H-6', CH_2CH_3), 3.74 (1H, dat, J_{at} 4.2 Hz, *J* 5.5 Hz, H-5), 3.80 (1H, ddd, $J_{1,2}$ 3.0 Hz, $J_{1,4a}$ 5.9 Hz, $J_{1,4a'}$ 7.3 Hz, H-1), 3.91 (1H, dd, $J_{2,3}$ 5.1 Hz, $J_{3,4}$ 8.6 Hz, H-3), 4.23, 4.47 (2H, 2 × d, *J* 11.2 Hz, $PhCH_2$), 4.40, 4.73 (2H, 2 × d, *J* 11.8 Hz, $PhCH_2$), 4.48, 4.54 (2H, 2 × d, *J* 12.1 Hz, $PhCH_2$), 5.16 (1H, dd, $J_{1,2}$ 3.1 Hz, $J_{2,3}$ 5.0 Hz, H-2), 7.23–7.34 (15H, m, Ar-H); δ_C (100 MHz, $CDCl_3$) 15.6 (q, CH_2CH_3), 27.8 (t, C-4a), 43.5 (d, C-4), 65.1 (t, CH_2CH_3), 72.7, 72.8, 73.0, 73.5 (4 × t, C-6, 3 × $PhCH_2$), 75.7 (d, C-2), 77.0 (d, C-5), 79.1 (d, C-3), 81.2 (d, C-1), 127.5, 127.7, 127.9, 128.3, 128.4, 128.5, 128.5 (7 × d, Ar-*C*H), 138.3, 138.5, 139.1 (3 × s, Ar-*C*), 170.5 (s, C=O).

Similarly, the regioisomeric β-*galacto* 2-*O*-ethyl ether (2 mg, 4 µmol) was converted by a similar procedure into its acetate (1 mg, 48%), an oil; δ_H (400 MHz, $CDCl_3$) 1.21 (3H, t, *J* 7.0 Hz, CH_2CH_3), 1.68 (1H, m, H-4a), 2.03–2.11 (4H, m, H-4a', C(O)CH_3), 2.31 (1H, m, H-4), 3.46–3.75 (6H, m, H-3, H-5, H-6, H-6', CH_2CH_3), 3.79 (1H, m, H-2), 4.36, 4.69 (2H, 2 × d, *J* 11.6 Hz, $PhCH_2$), 4.41, 4.63 (2H, 2 × d, *J* 11.7 Hz, $PhCH_2$), 4.47, 4.53 (2H, 2 × d, *J* 12.1 Hz, $PhCH_2$), 4.94 (1H, m, H-1), 7.24–7.34 (15H, m, Ar-H).

Methyl 3,5,6-tri-*O*-benzyl-4a-carba-α-D-talofuranosyl-(1→3)-2-*O*-benzyl-4,6-*O*-benzylidene-α-D-mannopyranoside (11)

Epoxide **4** (188 mg, 0.437 mmol) and *manno* alcohol **10** (712 mg, 1.91 mmol) were dissolved in CH_2Cl_2 (1.5 mL) at RT under a N_2 atmosphere. $BF_3 \cdot Et_2O$ (28 µL, 0.22 mmol) was dissolved in dry CH_2Cl_2 (2.0 mL), and 400 µL (0.044 mmol $BF_3 \cdot Et_2O$) of this solution transferred to the colourless reaction mixture, which instantly turned pale yellow. After 5 min, TLC (toluene–EtOAc, 6:1) showed formation of a major product (R_f 0.3), unreacted alcohol (R_f 0.4) and consumption of epoxide (R_f 0.6). The reaction was quenched after 15 min by the addition of Et_3N (0.5 mL) and concentrated *in vacuo*. The crude product was purified by column chromatography (pentane–EtOAc, 7:2) to yield pseudodisaccharide **11** (200 mg, 57%) as an oil; $[\alpha]_D^{25}$ −23.7 (c, 1.0 in $CHCl_3$); δ_H (400 MHz, $CDCl_3$) 1.69 (1H, ddd, $J_{1,4a}$ 5.0 Hz, $J_{4,4a}$ 8.9 Hz, $J_{4a,4a'}$ 13.7 Hz, H-4a^{II}), 2.12 (1H, ddd, $J_{1,4a'}$ 7.2 Hz, $J_{4,4a'}$ 9.1 Hz, $J_{4a,4a'}$ 13.5 Hz, H-4a'^{II}), 2.21 (1H, m, H-4^{II}), 3.34 (3H, s, CH_3), 3.51–3.58 (2H, m, H-6^{II}, H-6'^{II}), 3.69 (1H, aq, J 5.0 Hz, H-5^{II}), 3.76–3.81 (3H, m, H-2^{I}, H-5^{I}, H-3^{II}), 3.86 (1H, at, J 10.0 Hz, H-6^{I}), 3.95–4.13 (4H, m, H-3^{I}, H-4^{I}, H-1^{II}, H-2^{II}), 3.98, 4.22 (2H, 2 × d, J 11.3 Hz, PhCH_2), 4.25 (1H, dd, $J_{5,6'}$ 4.4 Hz, $J_{6,6'}$ 9.8 Hz, H-6'^{I}), 4.41, 4.74 (2H, 2 × d, J 11.8 Hz, PhCH_2), 4.48, 4.53 (2H, 2 × d, J 12.1 Hz, PhCH_2), 4.65, 4.80 (2H, 2 × d, J 12.2 Hz, PhCH_2), 4.68 (1H, d, $J_{1,2}$ 1.4 Hz, H-1^{I}), 5.57 (1H, s, PhC*H*), 7.12–7.48 (25H, m, Ar-H); δ_C (100 MHz, $CDCl_3$) 28.7 (t, C-4a^{II}), 43.1 (d, C-4^{II}), 54.9 (q, CH_3), 64.4 (d, C-5^{I}), 69.0 (t, C-6^{I}), 72.1, 72.6, 72.9, 73.5, 74.0 (5 × t, C-6^{II}, 4 × Ph*C*H_2), 74.0, 75.8, 78.2, 83.1 (4 × d, C-3^{I}, C-4^{I}, C-1^{II}, C-2^{II}), 76.2 (d, C-2^{I}), 78.0 (d, C-5^{II}), 80.9 (d, C-3^{II}), 100.9 (d, C-1^{I}), 102.1 (d, Ph*C*H), 126.3, 127.5, 127.7, 127.8, 127.9, 127.9, 128.1, 128.2, 128.3, 128.4, 128.5, 128.6, 129.2 (13 × d, Ar-CH), 137.9, 138.0, 138.3, 138.4, 139.1 (5 × s, 5 × Ar-C); IR 3500 (OH) cm^{-1}; HRMS Calcd. For $C_{49}H_{54}NaO_{10}$ (MNa^+) 825.3609. Found 825.3640.

Unreacted *manno* alcohol **10** (411 mg, 1.10 mmol) was recovered.

A by-product **13** (up to 33%) was also isolated; δ_H (400 MHz, $CDCl_3$) 1.38 (1H, d, $J_{4a,4a'}$ 12.8 Hz, H-4a), 1.96 (1H, ddd, $J_{1,4a'}$ 2.9 Hz, $J_{4,4a'}$ 6.1 Hz, $J_{4a,4a'}$ 12.7 Hz, H-4a'), 2.66 (1H, m, H-4), 3.05 (1H,

at, J 11.0 Hz, H-6), 3.29 (1H, bs, OH), 3.55 (1H, ddd, $J_{4,5}$ 3.4 Hz, $J_{5,6'}$ 6.6 Hz, $J_{5,6}$ 10.1 Hz, H-5), 3.86 (1H, dd, $J_{5,6'}$ 6.4 Hz, $J_{6,6'}$ 11.6 Hz, H-6'), 4.02–4.23 (3H, m, H-1, H-2, H-3), 4.44, 4.55 (2H, 2 × d, J 11.9 Hz, PhCH_2), 4.61 (2H, s, PhCH_2), 7.26–7.37 (10H, m, Ar-H); δ_C (100 MHz, $CDCl_3$) 31.7 (t, C-4a), 42.9 (d, C-4), 65.5 (t, C-6), 70.7, 73.3 (2 × t, 2 × PhCH$_2$), 73.8, 73.8, 77.4, 79.8 (4 × d, C-1, C-2, C-3, C-5), 127.6, 127.9, 128.1, 128.2, 128.6, 128.7 (6 × d, Ar-CH), 137.4, 138.3 (2 × s, 2 × Ar-C); *m/z* (ES^+) 363 ($M+Na^+$).

This by-product **13** was acetylated as described for **12** and characterised as its acetate **14**; $[\alpha]_D^{25}$ +10.1 (c, 0.32 in $CHCl_3$); δ_H (500 MHz, $CDCl_3$) 1.46 (1H, d, $J_{4a,4a'}$ 12.5 Hz, H-4a), 2.00–2.06 (4H, m, H-4a', C(O)CH_3), 2.68 (1H, m, H-4), 3.15 (1H, at, J 11.0 Hz, H-6), 3.56 (1H, ddd, $J_{5,6}$ 10.1 Hz, $J_{5,6'}$ 6.6 Hz, $J_{4,5}$ 3.4 Hz, H-5), 3.90 (1H, dd, $J_{5,6'}$ 6.5 Hz, $J_{6,6'}$ 11.8 Hz, H-6'), 4.13 (1H, s, H-1), 4.27 (1H, d, $J_{2,3}$ 6.1 Hz, H-3), 4.41, 4.53 (2H, 2 × d, J 12.0 Hz, (C-5)OPhCH_2), 4.43, 4.47 (2H, 2 × d, J 11.5 Hz, (C-3)OPhCH_2), 5.18 (1H, dd, $J_{1,2}$ 1.6 Hz, $J_{2,3}$ 6.1 Hz, H-2), 7.24–7.37 (10H, m, Ar-H); δ_C (125 MHz, $CDCl_3$) 20.9 (q, C(O)CH_3), 32.9 (t, C-4a), 43.3 (d, C-4), 65.5 (t, C-6), 70.6 (t, (C-5)OPhCH$_2$), 73.5 (d, C-5), 73.6 (t, (C-3)OPhCH_2), 76.3 (d, C-2), 77.5, 77.9 (2 × d, C-1, C-3), 127.6, 127.8, 127.9, 128.0, 128.5, 128.6 (6 × d, Ar-CH), 138.3, 138.4 (2 × s, 2 × Ar-C), 170.4 (s, C=O); IR 1722 (C=O) cm^{-1}; HRMS Calcd. For $C_{23}H_{26}NaO_5$ (MNa^+) 405.1669. Found 405.1672.

Methyl 2-*O*-acetyl-3,5,6-tri-*O*-benzyl-4a-carba-α-D-talofuranosyl-(1→3)-2-*O*-benzyl-4,6-*O*-benzylidene-α-D-mannopyranoside (12)

Pseudodisaccharide alcohol **11** (21 mg, 0.026 mmol) was dissolved in Ac_2O–pyridine, 1:1 (1 mL) and stirred at RT for 2 h, after which time TLC (toluene–EtOAc, 6:1) showed formation of a single product (R_f 0.7) and no remaining starting material (R_f 0.3). All the solvent was removed *in vacuo* and the crude product purified by column chromatography (toluene–EtOAc, 4:1) to give acetate **12** (20 mg, 91%) as an oil; $[\alpha]_D^{25}$ −26.0 (c, 1.0 in $CHCl_3$); δ_H (500 MHz, $CDCl_3$) 1.76 (1H, ddd, J 4.0 Hz, J 8.8 Hz, $J_{4a,4a'}$ 13.3 Hz, H-4a^{II}), 1.95 (3H, s, C(O)CH_3), 2.13 (1H, ddd, $J_{1,4a'}$ 7.5 Hz, $J_{4,4a'}$ 9.5 Hz, $J_{4a,4a'}$ 13.7 Hz, H-4a'^{II}), 2.23 (1H, aqd, J_{aq} 9.1 Hz, J 3.8 Hz, H-4^{II}), 3.36 (3H, s, OCH_3), 3.50–

3.56 (2H, m, H-6^{II}, H-6'^{II}), 3.71–3.89 (6H, m, H-2^{I}, H-5^{I}, H-6^{I}, H-3^{II}, H-5^{II}, PhC*H*H'), 3.97 (1H, dd, $J_{2,3}$ 3.2 Hz, $J_{3,4}$ 10.1 Hz, H-3^{I}), 4.04 (1H, m, H-1^{II}), 4.11 (1H, at, *J* 9.5 Hz, H-4^{I}), 4.20 (1H, d, *J* 11.2 Hz, PhCH*H'*), 4.25 (1H, dd, $J_{5,6}$ 4.3 Hz, $J_{6,6'}$ 9.7 Hz, H-6'^{I}), 4.37, 4.72 (2H, 2 × d, *J* 11.8 Hz, PhCH_2), 4.47, 4.52 (2H, 2 × d, *J* 12.1 Hz, PhCH_2), 4.61, 4.78 (2H, 2 × d, *J* 12.1 Hz, PhCH_2), 4.68 (1H, d, $J_{1,2}$ 1.3 Hz, H-1^{I}), 5.32 (1H, dd, *J* 2.1 Hz, *J* 4.2 Hz, H-2^{II}), 5.55 (1H, s, PhC*H*), 7.07–7.47 (25H, m, Ar-H); δ_C (125 MHz, $CDCl_3$) 21.1 (q, C(O)CH_3), 28.6 (t, C-4a^{II}), 43.3 (d, C-4^{II}), 55.0 (q, OCH_3), 64.3 (d, C-5^{I}), 69.0 (t, C-6^{I}), 72.4, 73.0, 73.5, 74.1 (4 × t, 4 × PhCH_2), 72.8 (t, C-6^{II}), 75.4 (d, C-2^{II}), 75.6 (d, C-3^{I}), 76.4, 77.3, 79.4 (3 × d, C-2^{I}, C-3^{II}, C-5^{II}), 78.3 (d, C-4^{I}), 80.7 (d, C-1^{II}), 100.9 (d, C-1^{I}), 102.1 (d, Ph*C*H), 126.4, 127.5, 127.7, 127.7, 127.7, 127.9, 128.2, 128.3, 128.3, 128.3, 128.5, 129.1 (12 × d, Ar-CH), 137.9, 138.3, 138.3, 138.5, 139.2 (5 × s, 5 × Ar-C), 170.4 (s, C=O); IR 1739 (C=O) cm^{-1}; HRMS Calcd. For $C_{51}H_{56}NaO_{11}$ (MNa^+) 867.3715. Found 867.3715.

3,5,6-Tri-*O*-benzyl-1-*O*-ethyl-4a-carba-α-D-*lyxo*-hexofuranose-2-ulose (15)

A cold (−60 °C) solution of Me_2SO (196 μL, 2.76 mmol) in CH_2Cl_2 (3 mL) was transferred to a solution of oxalyl chloride (118 μL, 1.36 mmol) in CH_2Cl_2 (3 mL) under a N_2 atmosphere at −60 °C. The reaction mixture was stirred for 30 min. Alcohol **8** (102 mg, 0.214 mmol) was dissolved in CH_2Cl_2 (4 mL) and transferred to the reaction vessel. The reaction was left to stir for 45 min at −60 °C. Et_3N (505 μL, 3.64 mmol) was added, the reaction mixture allowed to reach RT and left to stir for a further 30 min. After this time, TLC (toluene–EtOAc, 9:1) showed formation of a single product (R_f 0.6). CH_2Cl_2 (10 mL) was added and the reaction mixture washed successively with water (15 mL) and brine (2 × 15 mL), and the aqueous phases were extracted with CH_2Cl_2 (3 × 10 mL). The combined organic extracts were dried ($MgSO_4$), filtered and concentrated *in vacuo*. The crude product was purified by flash chromatography (pentane–EtOAc, 5:1) to give ketone **15** (89 mg, 88%) as an oil; δ_H (400 MHz, $CDCl_3$) 1.23 (3H, t, *J* 7.0 Hz, CH_2CH_3), 1.69 (1H, aq, *J* 12.5 Hz, H-4a), 2.17 (1H, m, H-4), 2.28 (1H, ddd, $J_{4a,4a'}$ 12.3 Hz, *J* 6.3 Hz, *J* 8.4 Hz, H-4a'), 3.54–3.66 (3H, m, C*H*H'CH_3, H-6, H-6'), 3.76–3.84 (4H, m, CH*H'*CH_3, H-1, H-3, H-5), 4.24, 4.65 (2H, 2 × d, *J*

11.7 Hz, PhCH_2), 4.50, 4.96 (2H, 2 × d, *J* 11.4 Hz, PhCH_2), 4.50, 4.57 (2H, 2 × d, *J* 12.0 Hz, PhCH_2), 7.20–7.38 (15H, m, Ar-H); δ_C (100 MHz, $CDCl_3$) 15.4 (q, CH_2CH_3), 24.9 (t, C-4a), 39.4 (d, C-4), 66.2 (t, CH_2CH_3), 71.3 (t, C-6), 72.7, 73.0, 73.5 (3 × t, 3 × PhCH_2), 75.6, 78.4, 79.2 (3 × d, C-1, C-3, C-5), 127.8, 127.8, 127.9, 127.9, 128.1, 128.4, 128.5, 128.5, 128.6 (9 × d, Ar-CH), 137.7, 138.2, 138.5 (3 × s, Ar-C), 214.4 (s, C-2); HRMS Calcd. For $C_{30}H_{34}NaO_5$ (MNa^+) 497.2298. Found 497.2292.

Methyl 3,5,6-tri-*O*-benzyl-1-*O*-ethyl-4a-carba-α-D-*lyxo*-hexofuranosyl-2-ulose-(1→3)-2-*O*-benzyl-4,6-*O*-benzylidene-α-D-mannopyranoside (16)

A cold (−60 °C) solution of Me_2SO (98 μL, 1.29 mmol) in CH_2Cl_2 (1 mL) was transferred to a solution of oxalyl chloride (59 μL, 0.64 mmol) in CH_2Cl_2 (1 mL) under a N_2 atmosphere at −60 °C. The reaction mixture was stirred for 30 min. Alcohol **11** (129 mg, 0.161 mmol) was dissolved in CH_2Cl_2 (3 mL) and transferred to the reaction vessel. The reaction was left to stir for 45 min at −60 °C. Et_3N (251 μL, 1.93 mmol) was added and the reaction allowed to reach RT and left to stir for a further 30 min. After this time, TLC (toluene–EtOAc, 5:1) showed the complete consumption of starting material (R_f 0.4) and the formation of a single product (R_f 0.8). CH_2Cl_2 (5 mL) was added and the reaction mixture washed successively with water (10 mL) and brine (2 × 10 mL) and the aqueous phases were extracted with CH_2Cl_2 (3 × 10 mL). The combined organic extracts were dried ($MgSO_4$), filtered and concentrated *in vacuo*. The crude product was purified by flash chromatography (toluene–EtOAc, 8:1) to give ketone **16** (88 mg, 68%) as an oil; δ_H (400 MHz, $CDCl_3$) 1.68 (1H, aq, *J* 12.2 Hz, H-4a^{II}), 2.09 (1H, m, H-4^{II}), 2.28 (1H, ddd, *J* 6.4 Hz, *J* 8.4 Hz, $J_{4a,4a'}$ 12.3 Hz, H-4a'^{II}), 3.36 (3H, s, OCH_3), 3.51 (1H, dd, $J_{5,6}$ 5.2 Hz, $J_{6,6'}$ 9.9 Hz, H-6^{II}), 3.60 (1H, dd, $J_{5,6'}$ 6.0 Hz, $J_{6,6'}$ 9.9 Hz, H-6'^{II}), 3.75–3.81 (4H, m, H-2^{I}, H-5^{I}, H-3^{II}, H-5^{II}), 3.90 (1H, at, *J* 10.3 Hz, H-6^{I}), 4.06 (1H, dd, $J_{2,3}$ 3.2 Hz, $J_{3,4}$ 9.9 Hz, H-3^{I}), 4.25–4.33 (4H, m, H-4^{I}, H-6'^{I}, H-1^{II}, PhC*H*H'), 4.42, 4.92 (2H, 2 × d, *J* 11.4 Hz, PhCH_2), 4.48, 4.54 (2H, 2 × d, *J* 12.1 Hz, PhCH_2), 4.66

(1H, d, J 11.6 Hz, PhCHH'), 4.69 (1H, d, $J_{1,2}$ 1.6 Hz, H-1^{I}), 4.72, 4.86 (2H, 2 × d, J 12.1 Hz, PhCH_2), 5.64 (1H, s, PhCH), 7.21–7.48 (25H, m, Ar-H); δ_C (100 MHz, $CDCl_3$) 25.0 (t, C-4a^{II}), 39.3 (d, C-4^{II}), 54.9 (q, OCH_3), 64.0, 75.6, 76.9, 78.3 (4 × d, C-2^{I}, C-5^{I}, C-3^{II}, C-5^{II}), 68.9 (t, C-6^{I}), 71.5 (t, C-6^{II}), 72.6, 73.0, 73.5, 74.0 (4 × t, 4 × PhCH_2), 75.1 (d, C-3^{I}), 79.2, 79.5 (2 × d, C-4^{I}, C-1^{II}), 100.8 (d, C-1^{I}), 101.6 (d, PhCH), 126.1, 127.8, 127.8, 127.8, 128.0, 128.0, 128.3, 128.4, 128.5, 128.6, 128.6, 128.9 (12 × d, Ar-CH), 137.7, 137.8, 138.1, 138.3, 138.6 (5 × s, 5 × Ar-C), 212.5 (s, C-2^{II}); IR 1758 (C=O) cm^{-1}; HRMS Calcd. For $C_{49}H_{52}NaO_{10}$ (MNa^+) 823.3453. Found 823.3446.

3,5,6-Tri-*O*-benzyl-1-*O*-ethyl-4a-carba-α-D-galactofuranose (17)

Ketone **15** (49 mg, 0.103 mmol) was dissolved in THF (2 mL) under a N_2 atmosphere and the mixture was cooled to 0 °C. L-Selectride (1 M in THF, 155 µL, 0.155 mmol) was added, and the reaction mixture stirred at RT for 18 h, after which TLC (toluene–EtOAc, 5:1) showed complete consumption of starting material (R_f 0.8) and formation of a major product (R_f 0.5) and a minor product (R_f 0.45). The reaction mixture was diluted with THF (5 mL), quenched by the addition of NH_4Cl (satd. aq., 0.5 mL), and washed with H_2O (2 × 5 mL). The aqueous phase was extracted with CH_2Cl_2 (3 × 10 mL). The combined organic extracts were dried ($MgSO_4$), filtered and concentrated *in vacuo*. The crude product was purified by column chromatography (pentane–EtOAc, 4:1) to yield α-*galacto* alcohol **17** (39 mg, 78%) as an oil; δ_H (400 MHz, $CDCl_3$) 1.23 (3H, t, 7.0 Hz, CH_2CH_3), 1.75 (1H, ddd, $J_{1,4a}$ 7.4 Hz, $J_{4,4a}$ 9.2 Hz, $J_{4a,4a'}$ 12.4 Hz, H-4a), 1.97–2.11 (2H, m, H-4, H-4a'), 2.85 (1H, d, $J_{OH,2}$ 5.1 Hz, OH-2), 3.44–3.60 (4H, m, CH_2CH$_3$, H-6, H-6'), 3.66 (1H, dd, J 3.8 Hz, J 6.3 Hz, H-3), 3.70 (1H, aq, J 5.2 Hz, H-5), 3.81 (1H, dat, J_{at} 6.0 Hz, $J_{1,4a}$ 7.4 Hz, H-1), 4.04 (1H, m, H-2), 4.44 (1H, d, J 11.7 Hz, PhCHH'), 4.45 (1H, d, J 11.5 Hz, PhCHH'), 4.48, 4.54 (2H, 2 × d, J 12.1 Hz, PhCH_2), 4.71 (1H, d, J 11.6 Hz, PhCHH'), 4.73 (1H, d, J 11.5 Hz, PhCHH'), 7.24–7.37 (15H, m, Ar-H); δ_C (100 MHz, $CDCl_3$) 15.5 (q, CH_2CH_3), 28.3 (t, C-4a), 43.2 (d, C-4), 65.1 (t, CH$_2$CH$_3$), 72.0, 72.4, 73.1, 73.5 (4 × t, 3 × PhCH_2, C-6), 75.8 (d, C-2), 78.5, 78.5 (2 × d, C-1, C-5), 85.9 (d, C-3), 127.6, 127.7, 127.8, 127.9, 128.0, 128.1, 128.4, 128.5, 128.5 (9 × d, Ar-CH), 138.4, 138.5, 138.8

(3 × s, 3 × Ar-C); IR 3482 (OH) cm^{-1}; HRMS Calcd. For $C_{30}H_{36}NaO_5$ (MNa^+) 499.2455. Found 499.2466.

A mixed fraction (5 mg, 10%) consisting of a 1:1 epimeric mixture of α-*galacto* **17** and α-*talo* **8** alcohols was also isolated.

Methyl 3,5,6-tri-*O*-benzyl-4a-carba-α-D-galactofuranosyl-(1→3)-2-*O*-benzyl-4,6-*O*-benzylidene-α-D-mannopyranoside (18)

Ketone **16** (87 mg, 0.11 mmol) was dissolved in THF (4 mL) under a N_2 atmosphere and the mixture was cooled to 0 °C. L-Selectride (1 M in THF, 163 µL, 0.163 mmol) was added, and the reaction mixture stirred at RT for 1 h, after which time TLC (toluene–EtOAc, 8:1) showed complete consumption of starting material (R_f 0.7) and formation of a major product (R_f 0.3). The reaction was quenched by the addition of NH_4Cl (satd. aq., 1 mL), washed with H_2O (2 × 5 mL). The aqueous phase was extracted with CH_2Cl_2 (3 × 10 mL). The combined organic extracts were dried ($MgSO_4$), filtered and concentrated *in vacuo*. The crude product was purified by column chromatography (toluene–EtOAc, 12:1) to yield the α-*galacto* pseudodisaccharide **18** (58 mg, 66%) as an oil; $[\alpha]_D^{25}$ −25.2 (c, 1.0 in $CHCl_3$); δ_H (500 MHz, $CDCl_3$) 1.86 (1H, m, H-4a^{II}), 1.98–2.08 (2H, m, H-4a'^{II}, H-4^{II}), 3.27 (1H, bs, OH-2), 3.35 (3H, s, OCH_3), 3.52 (1H, dd, $J_{5,6}$ 6.2 Hz, $J_{6,6'}$ 10.4 Hz, H-6^{II}), 3.56 (1H, dd, $J_{5,6'}$ 3.7 Hz, $J_{6,6'}$ 10.4 Hz, H-6'^{II}), 3.61 (1H, dd, *J* 3.1 Hz, *J* 6.2 Hz, H-3^{II}), 3.65 (1H, m, H-5^{II}), 3.78–3.83 (2H, m, H-2^{I}, H-5^{I}), 3.88 (1H, at, *J* 10.2 Hz, H-6^{I}), 3.94 (1H, dd, $J_{2,3}$ 3.2 Hz, $J_{3,4}$ 10.2 Hz, H-3^{I}), 3.98 (1H, m, H-1^{II}), 4.04 (1H, m, H-2^{II}), 4.18 (1H, at, *J* 9.7 Hz, H-4^{I}), 4.27 (1H, dd, $J_{5,6'}$ 4.5 Hz, $J_{6,6'}$ 10.0 Hz, H-6'^{I}), 4.29, 4.57 (2H, 2 × d, *J* 11.6 Hz, PhCH_2), 4.44 (1H, d, *J* 11.7 Hz, PhC*H*H'), 4.45, 4.50 (2H, 2 × d, *J* 12.1 Hz, PhCH_2), 4.67, 4.80 (2H, 2 × d, *J* 12.2 Hz, PhCH_2), 4.70–4.73 (2H, m, H-1^{I}, PhCH*H*'), 5.65 (1H, s, PhC*H*), 7.22–7.50 (25H, m, Ar-H); δ_C (125 MHz, $CDCl_3$) 30.2 (t, C-4a^{II}), 43.1 (d, C-4^{II}), 55.1 (q, OCH_3), 64.3, 75.9 (2 × d, C-2^{I}, C-5^{I}), 69.0 (t, C-6^{I}), 72.0, 72.4, 73.0, 73.4, 74.1 (5 × t, 4 × $PhCH_2$, C-6^{II}), 75.0 (d, C-2^{II}), 76.3 (d, C-3^{I}), 78.0 (d, C-4^{I}), 78.9 (d, C-1^{II}), 79.5 (d, C-5^{II}), 85.4 (d, C-3^{II}), 100.7 (d, C-1^{I}), 102.3 (d, Ph*C*H), 126.2, 127.5,

127.7, 127.7, 127.9, 128.0, 128.1, 128.2, 128.3, 128.4, 128.5, 128.5, 128.6, 129.3 (14 × d, Ar-*C*H), 137.2, 138.0, 138.4, 138.5, 139.1 (5 × s, 5 × Ar-C); IR 3499 (OH) cm^{-1}; HRMS Calcd. For $C_{49}H_{54}NaO_{10}$ (MNa^+) 825.3609. Found 825.3606.
