# Supplementary material for: Carbasugar analogues of galactofuranosides: α-O-linked derivatives
Source: Beilstein J Org Chem. 2010 Nov 29;6:1127–31. doi: 10.3762/bjoc.6.129 (PMC3001987; doi:10.3762/bjoc.6.129)
Supplement: File 2 — NMR spectra. [file Beilstein_J_Org_Chem-06-1127-s002.pdf]

Supporting Information

for

Carbasugar analogues of galactofuranosides: α-*O*-linked derivatives

Jens Frigell[1] and Ian Cumpstey[*1,2]

Address: [1]Department of Organic Chemistry, The Arrhenius Laboratory, Stockholm University, Stockholm 106 91, Sweden and [2]Institut de Chimie des Substances Naturelles, CNRS, 91198 Gif-sur-Yvette, France, Tel.: +33 (0)1 69 82 30 78; fax: +33 (0)1 69 07 72 47

Email: Ian Cumpstey - ian.cumpstey@icsn.cnrs-gif.fr; ian.cumpstey@sjc.oxon.org

* Corresponding author

Contents

NMR spectra for new compounds

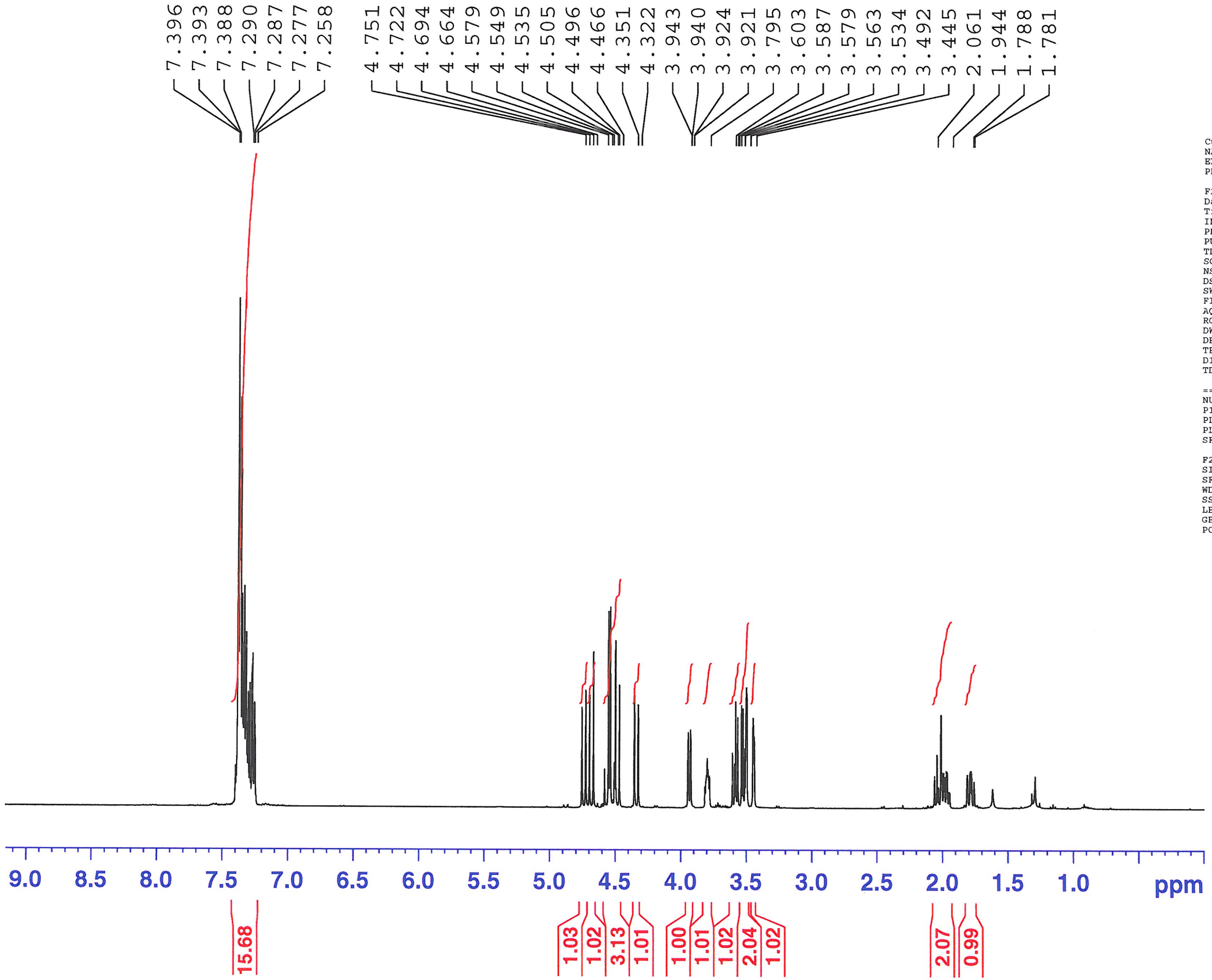

BRUKER

```
Current Data Parameters
NAME        JF-32-67-fr-9-19-epoxiso
EXPNO                     10
PROCNO                     1

F2 - Acquisition Parameters
Date_               20100201
Time                    8.20
INSTRUM                spect
PROBHD     5 mm PABBO BB-
PULPROG                 zg30
TD                     65536
SOLVENT                CDCl3
NS                        16
DS                         2
SWH                 8223.685 Hz
FIDRES              0.125483 Hz
AQ                 3.9846387 sec
RG                       256
DW                    60.800 usec
DE                      6.50 usec
TE                     296.6 K
D1                1.00000000 sec
TD0                        1

======== CHANNEL f1 ========
NUC1                      1H
P1                      8.90 usec
PL1                    -4.00 dB
PL1W             24.73352814 W
SFO1             400.1324710 MHz

F2 - Processing parameters
SI                     32768
SF               400.1300000 MHz
WDW                       EM
SSB                        0
LB                      0.30 Hz
GB                         0
PC                      1.00
```

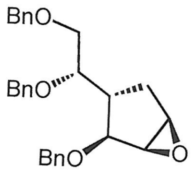

4

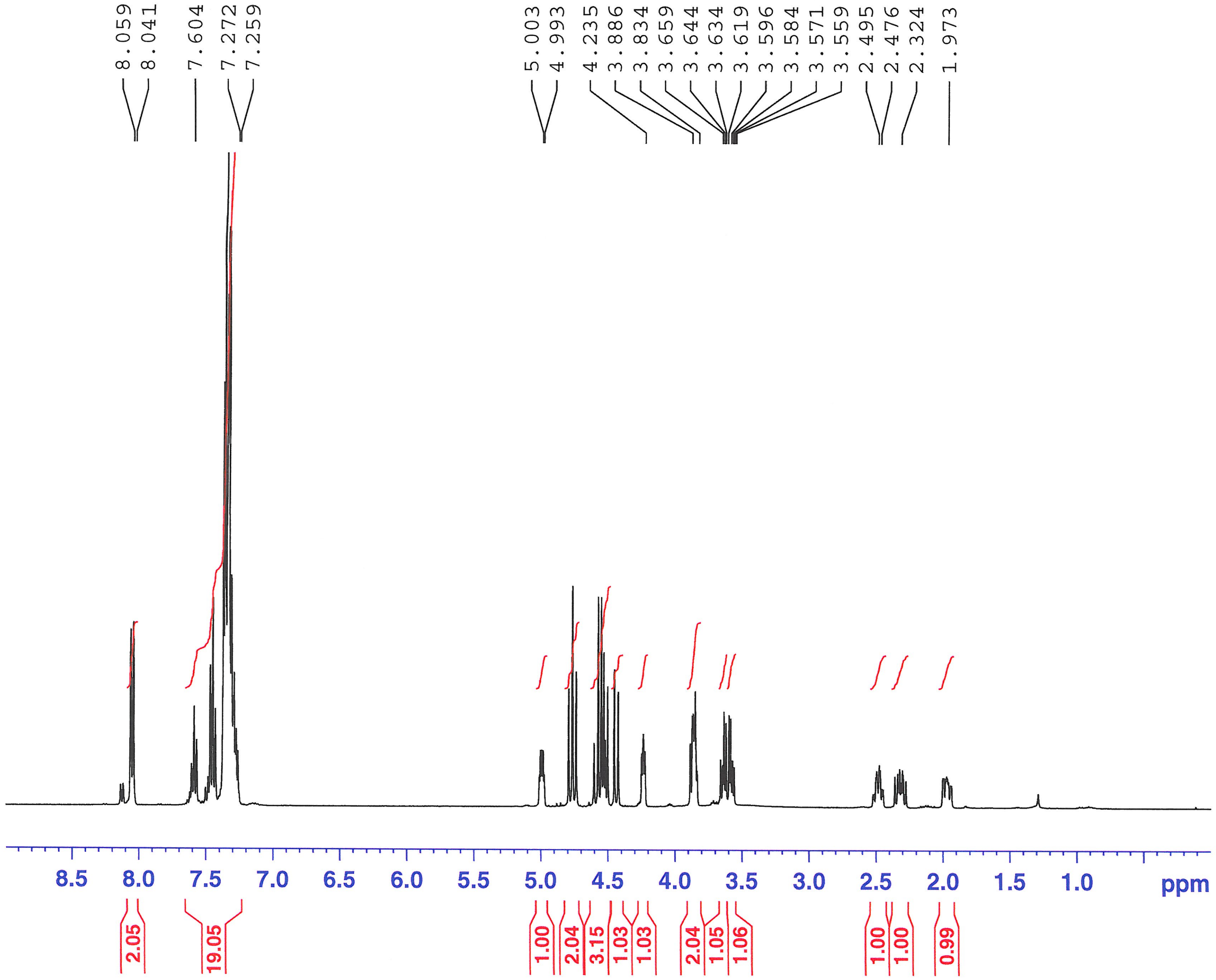

Current Data Parameters

NAME	JF-32-59-fr-29-42-sectry
EXPNO	10
PROCNO	1

F2 - Acquisition Parameters

Date_	20091214
Time	9.20
INSTRUM	spect
PROBHD	5 mm PABBO BB-
PULPROG	zg30
TD	65536
SOLVENT	CDCl3
NS	16
DS	2
SWH	8223.685 Hz
FIDRES	0.125483 Hz
AQ	3.9846387 sec
RG	144
DW	60.800 usec
DE	6.50 usec
TE	297.2 K
D1	1.00000000 sec
TD0	1

======== CHANNEL f1 ========

NUC1	1H
P1	8.90 usec
PL1	-4.00 dB
PL1W	24.73352814 W
SFO1	400.1324710 MHz

F2 - Processing parameters

SI	32768
SF	400.1300097 MHz
WDW	EM
SSB	0
LB	0.30 Hz
GB	0
PC	1.00

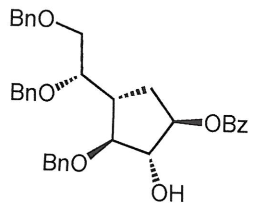

6

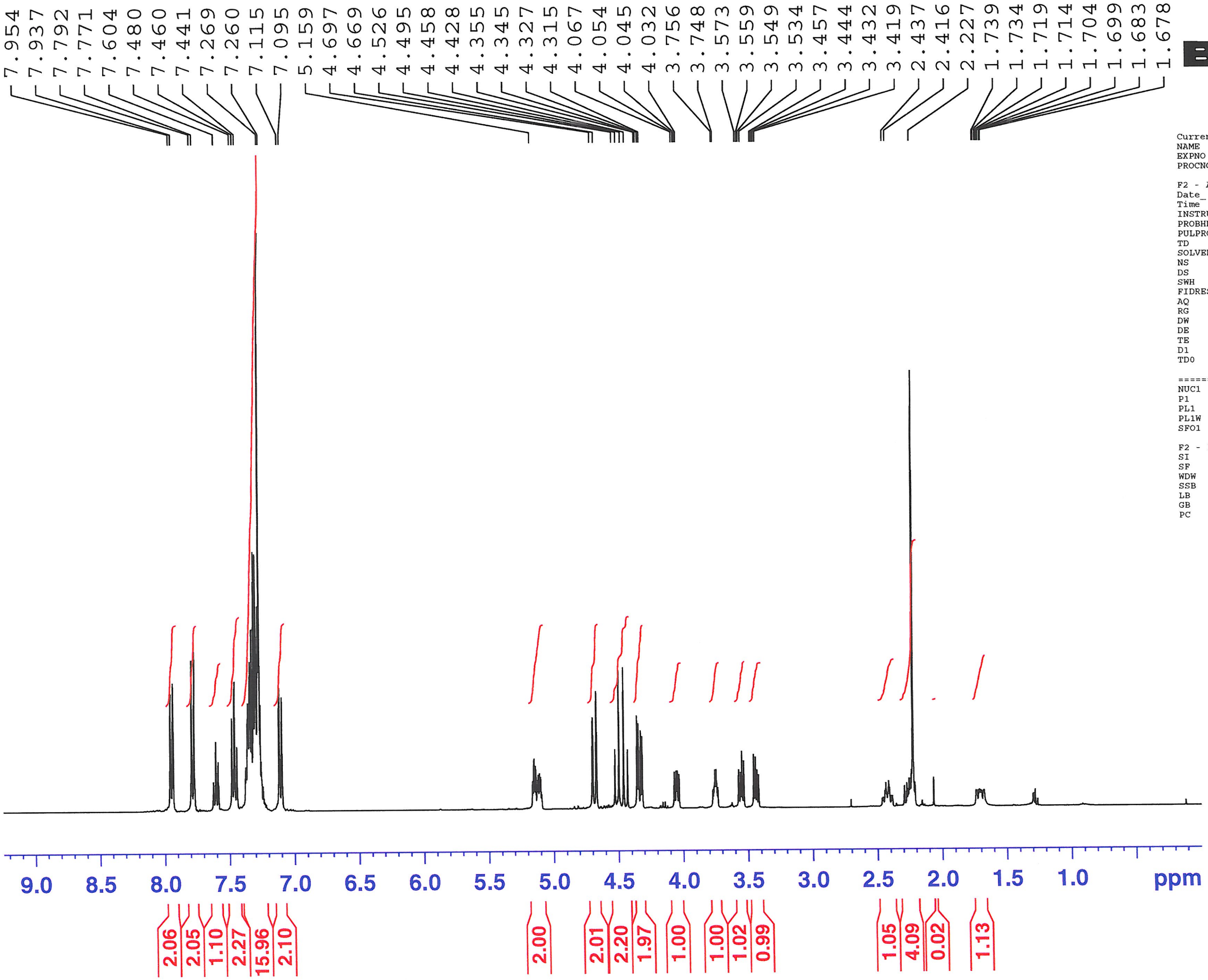

BRUKER

```
Current Data Parameters
NAME      JF-32-95-fr-21-38-BzOTsO
EXPNO                   10
PROCNO                   1

F2 - Acquisition Parameters
Date_             20100319
Time                 10.50
INSTRUM              spect
PROBHD    5 mm PABBO BB-
PULPROG               zg30
TD                   65536
SOLVENT              CDCl3
NS                      16
DS                       2
SWH               8223.685 Hz
FIDRES            0.125483 Hz
AQ               3.9846387 sec
RG                     101
DW                  60.800 usec
DE                    6.50 usec
TE                   296.9 K
D1              1.00000000 sec
TD0                      1

======== CHANNEL f1 ========
NUC1                    1H
P1                    8.90 usec
PL1                  -4.00 dB
PL1W           24.73352814 W
SFO1           400.1324710 MHz

F2 - Processing parameters
SI                   32768
SF             400.1300091 MHz
WDW                     EM
SSB                      0
LB                    0.30 Hz
GB                       0
PC                    1.00
```

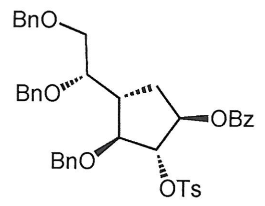

7

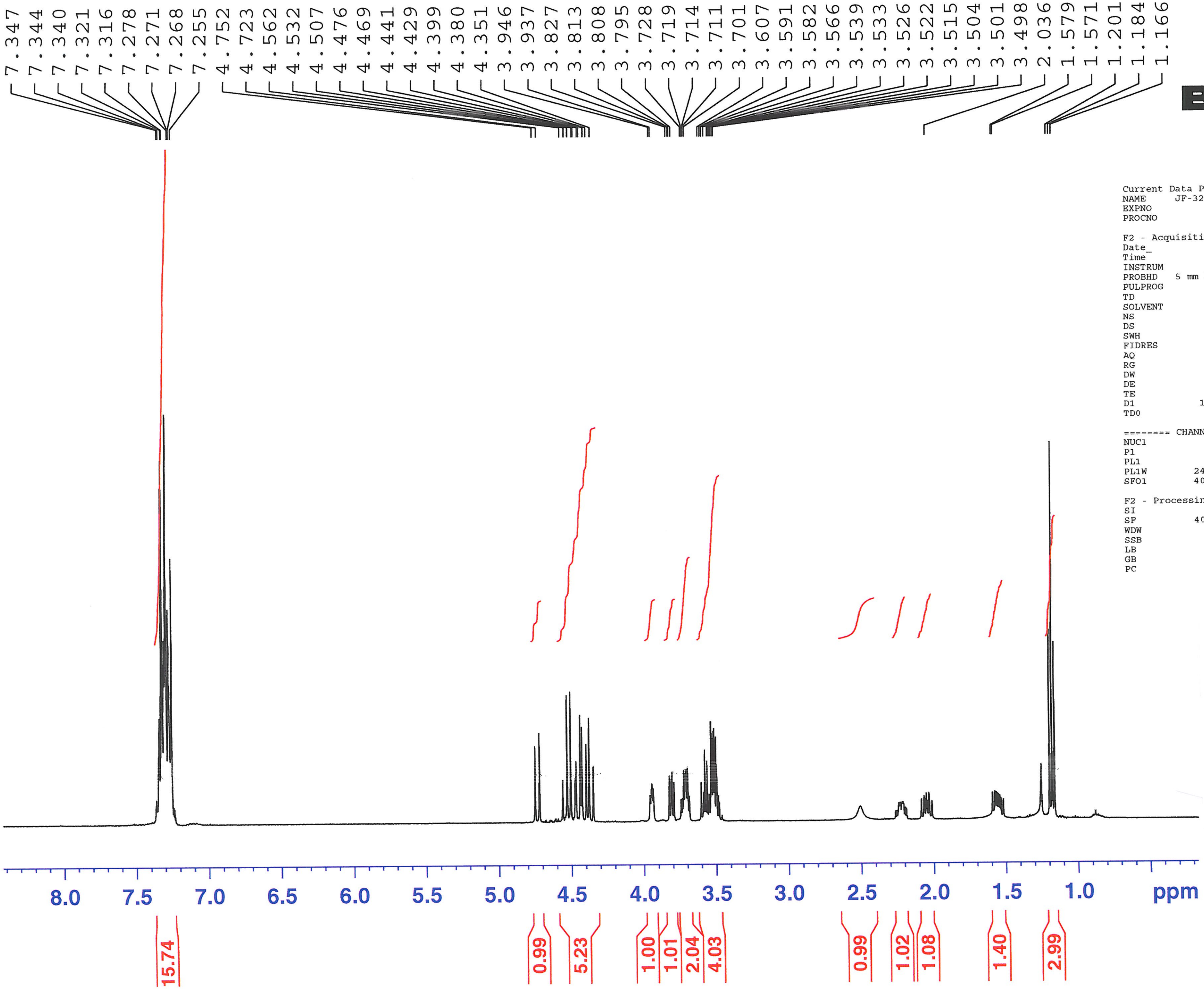

BRUKER

```
Current Data Parameters
NAME     JF-32-62-fr-11-18-ROEt
EXPNO                    10
PROCNO                    1

F2 - Acquisition Parameters
Date_              20091216
Time                   2.01
INSTRUM               spect
PROBHD   5 mm PABBO BB-
PULPROG                zg30
TD                    65536
SOLVENT               CDCl3
NS                       16
DS                        2
SWH                8223.685 Hz
FIDRES             0.125483 Hz
AQ                3.9846387 sec
RG                      362
DW                   60.800 usec
DE                     6.50 usec
TE                    297.2 K
D1               1.00000000 sec
TD0                       1

======== CHANNEL f1 ========
NUC1                     1H
P1                     8.90 usec
PL1                   -4.00 dB
PL1W            24.73352814 W
SFO1            400.1324710 MHz

F2 - Processing parameters
SI                    32768
SF              400.1300093 MHz
WDW                      EM
SSB                       0
LB                     0.30 Hz
GB                        0
PC                     1.00
```

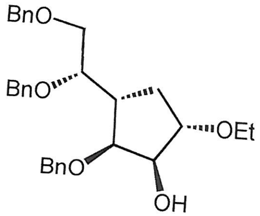

8

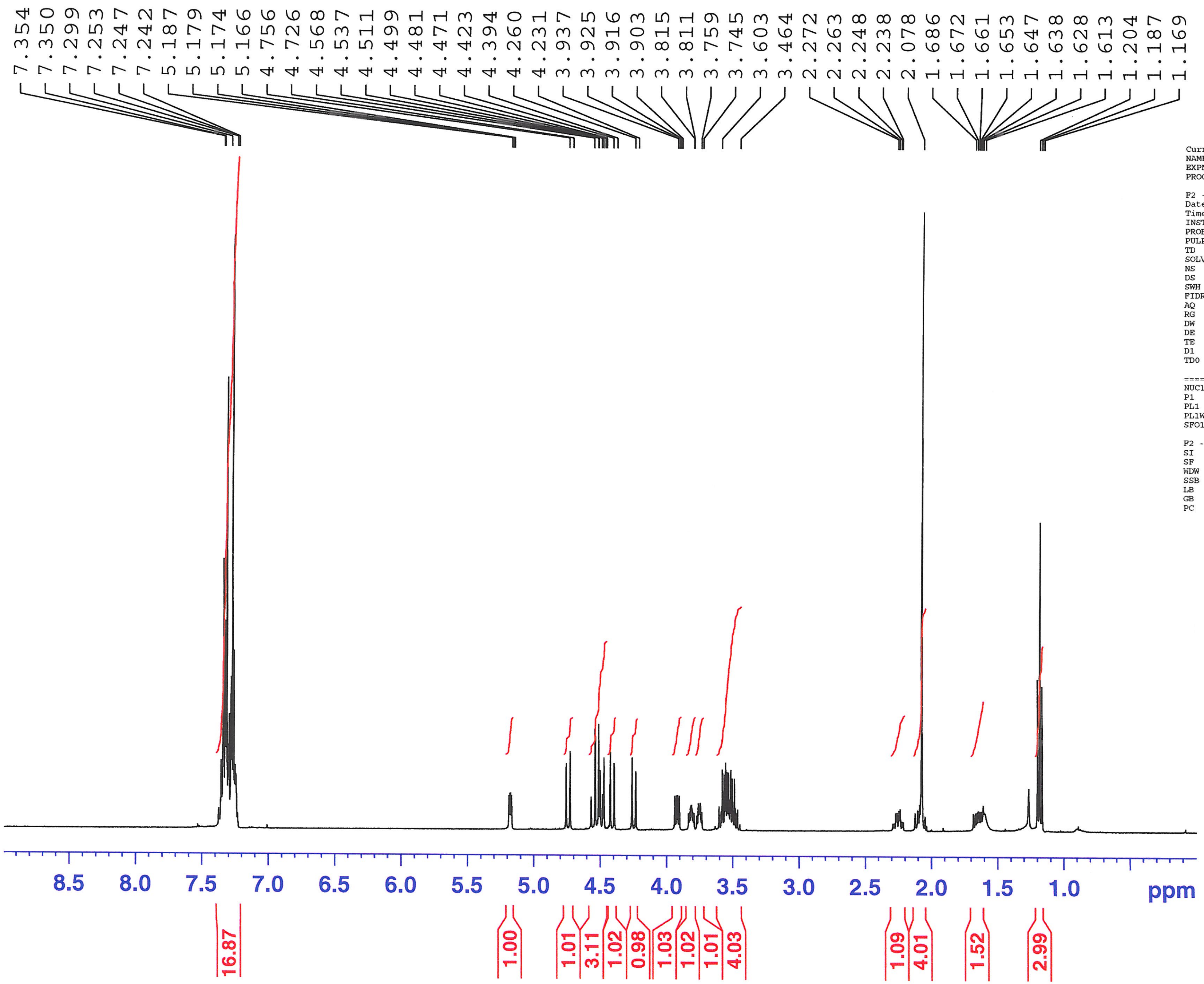

BRUKER

```
Current Data Parameters
NAME      JP-32-42-fr-7-11-Acetylated
EXPNO                   10
PROCNO                   1

F2 - Acquisition Parameters
Date_             20091106
Time                  5.21
INSTRUM              spect
PROBHD    5 mm PABBO BB-
PULPROG               zg30
TD                   65536
SOLVENT              CDCl3
NS                      16
DS                       2
SWH               8223.685 Hz
FIDRES            0.125483 Hz
AQ               3.9846387 sec
RG                     406
DW                  60.800 usec
DE                    6.50 usec
TE                   297.2 K
D1              1.00000000 sec
TD0                      1

======== CHANNEL f1 ========
NUC1                    1H
P1                    8.90 usec
PL1                  -4.00 dB
PL1W           24.73352814 W
SFO1           400.1324710 MHz

F2 - Processing parameters
SI                   32768
SF             400.1300049 MHz
WDW                     EM
SSB                      0
LB                    0.30 Hz
GB                       0
PC                    1.00
```

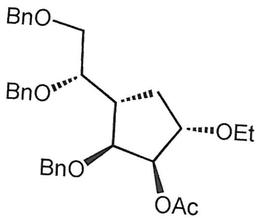

9

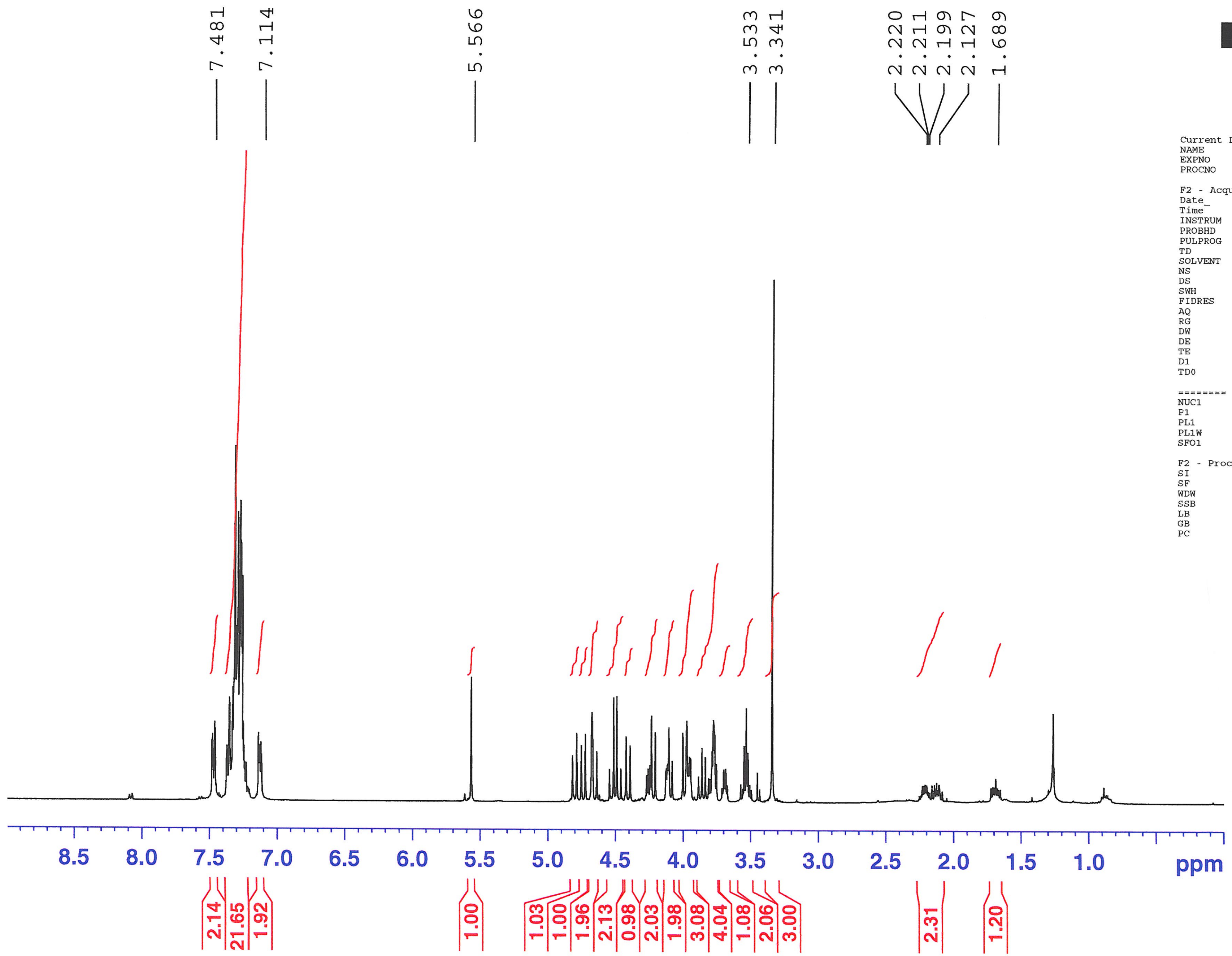

Current Data Parameters

NAME	JF-32-102-FL2-fr-38-51
EXPNO	10
PROCNO	1

F2 - Acquisition Parameters

Date_	20100422
Time	8.40
INSTRUM	spect
PROBHD	5 mm PABBO BB-
PULPROG	zg30
TD	65536
SOLVENT	CDCl3
NS	16
DS	2
SWH	8223.685 Hz
FIDRES	0.125483 Hz
AQ	3.9846387 sec
RG	287
DW	60.800 usec
DE	6.50 usec
TE	296.5 K
D1	1.00000000 sec
TD0	1

======== CHANNEL f1 ========

NUC1	1H
P1	8.90 usec
PL1	-4.00 dB
PL1W	24.73352814 W
SFO1	400.1324710 MHz

F2 - Processing parameters

SI	32768
SF	400.1300098 MHz
WDW	EM
SSB	0
LB	0.30 Hz
GB	0
PC	1.00

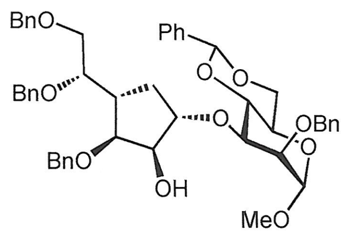

11

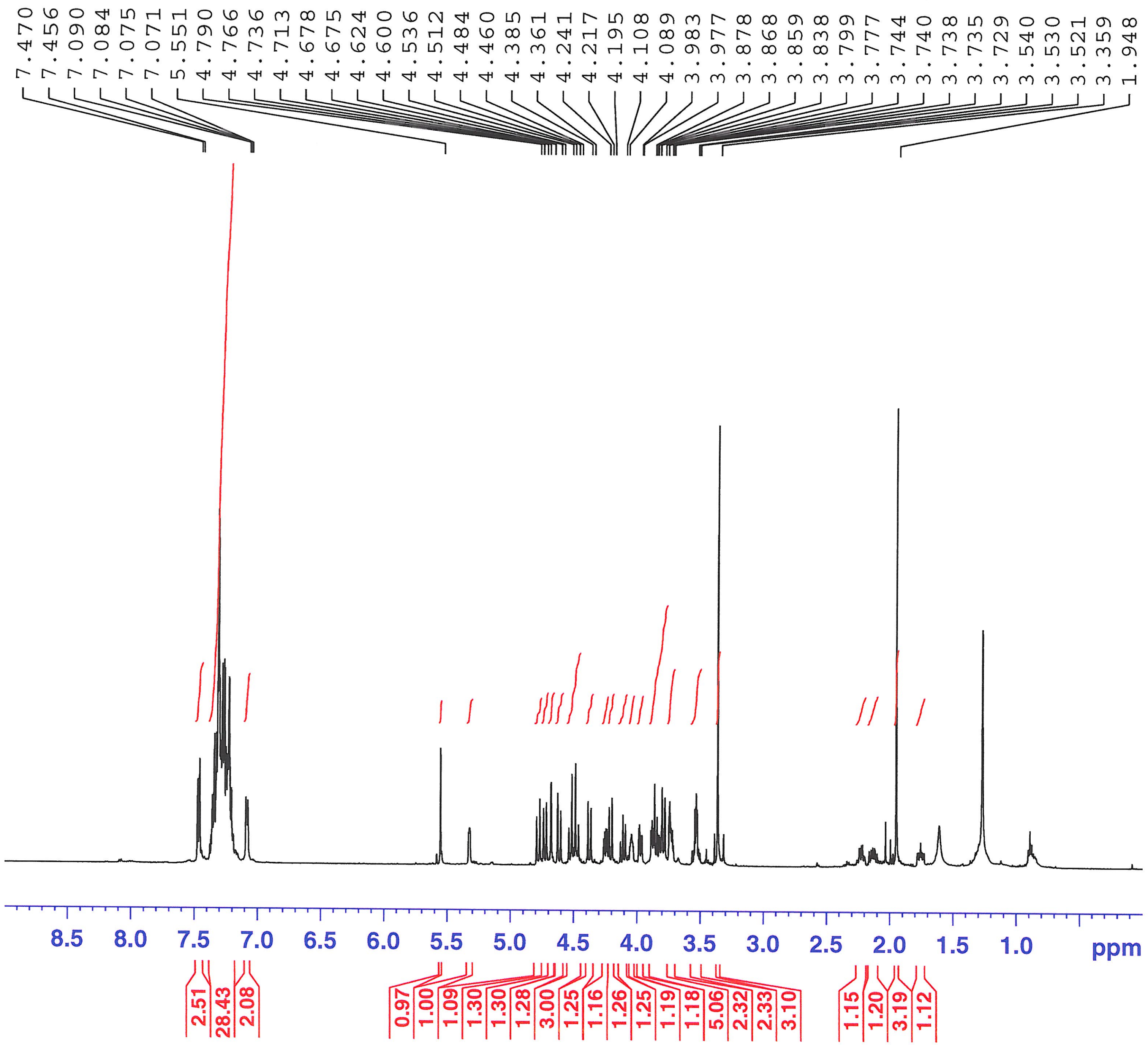

Current Data Parameters
NAME JF-38-19-acetylation
EXPNO 10
PROCNO 10

F2 - Acquisition Parameters
Date_ 20100823
Time 2.24
INSTRUM spect
PROBHD 5 mm PABBO BB-
PULPROG zg30
TD 65536
SOLVENT CDCl3
NS 16
DS 0
SWH 8012.820 Hz
FIDRES 0.122266 Hz
AQ 4.0894966 sec
RG 161
DW 62.400 usec
DE 6.00 usec
TE 298.2 K
D1 2.00000000 sec
TD0 1

======== CHANNEL f1 ========
NUC1 1H
P1 11.80 usec
PL1 2.00 dB
PL1W 15.76968765 W
SFO1 500.1325007 MHz

F2 - Processing parameters
SI 32768
SF 500.1300132 MHz
WDW EM
SSB 0
LB 0.30 Hz
GB 0
PC 1.00

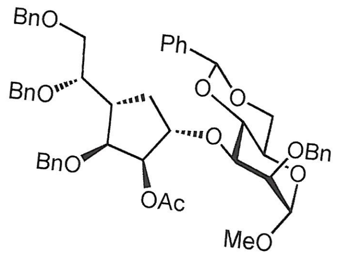

12

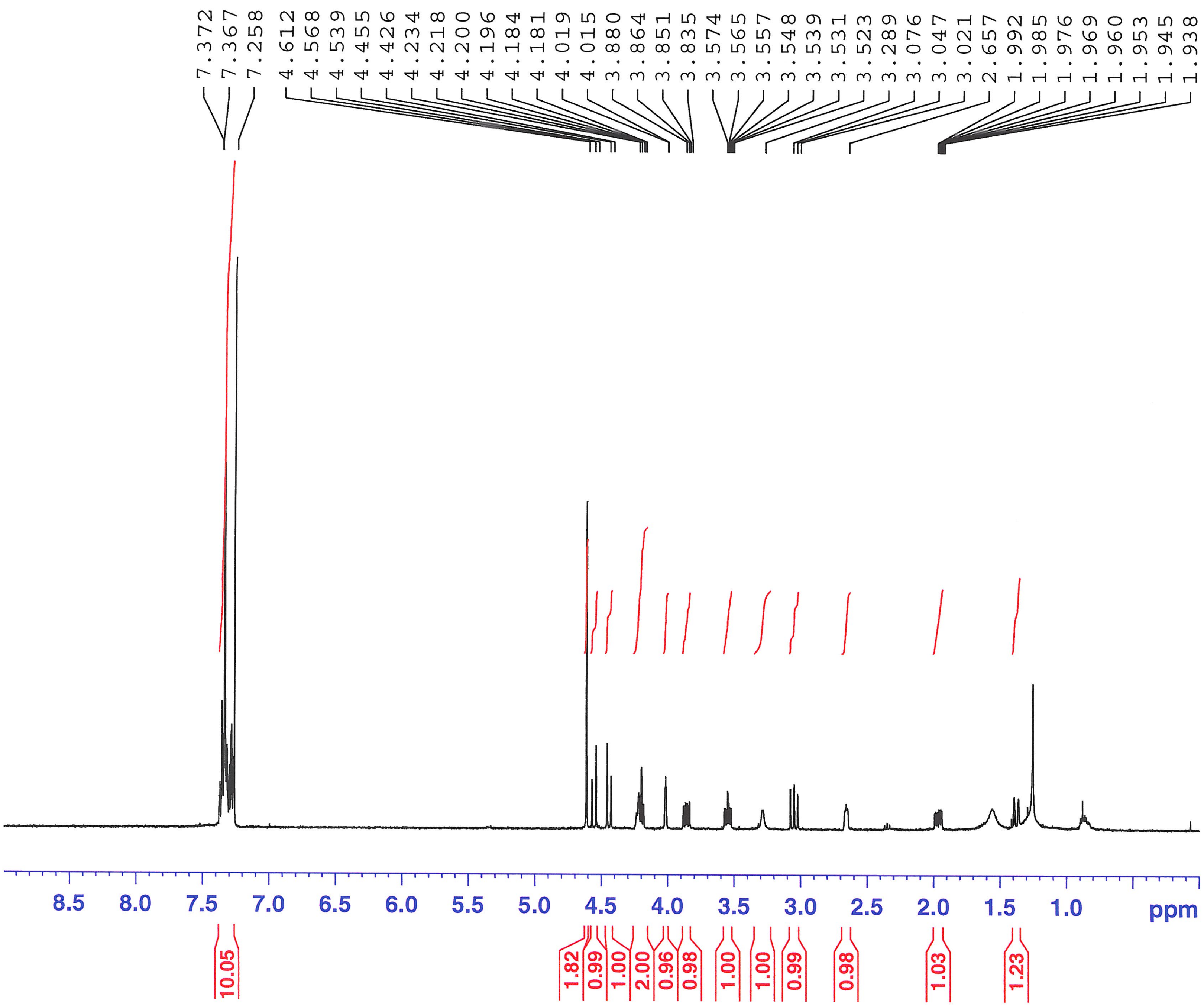


```
Current Data Parameters
NAME      JF-32-102-FL2-fr-27-34
EXPNO                    10
PROCNO                    1

F2 - Acquisition Parameters
Date_              20100422
Time                   8.34
INSTRUM               spect
PROBHD    5 mm PABBO BB-
PULPROG                zg30
TD                    65536
SOLVENT               CDCl3
NS                       16
DS                        2
SWH                8223.685 Hz
FIDRES             0.125483 Hz
AQ                3.9846387 sec
RG                      512
DW                   60.800 usec
DE                     6.50 usec
TE                    296.4 K
D1               1.00000000 sec
TD0                       1

======== CHANNEL f1 ========
NUC1                     1H
P1                     8.90 usec
PL1                   -4.00 dB
PL1W            24.73352814 W
SFO1            400.1324710 MHz

F2 - Processing parameters
SI                    32768
SF              400.1300103 MHz
WDW                      EM
SSB                       0
LB                     0.00 Hz
GB                        0
PC                     1.00
```

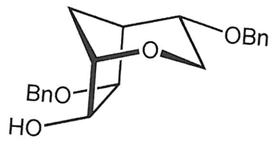

13

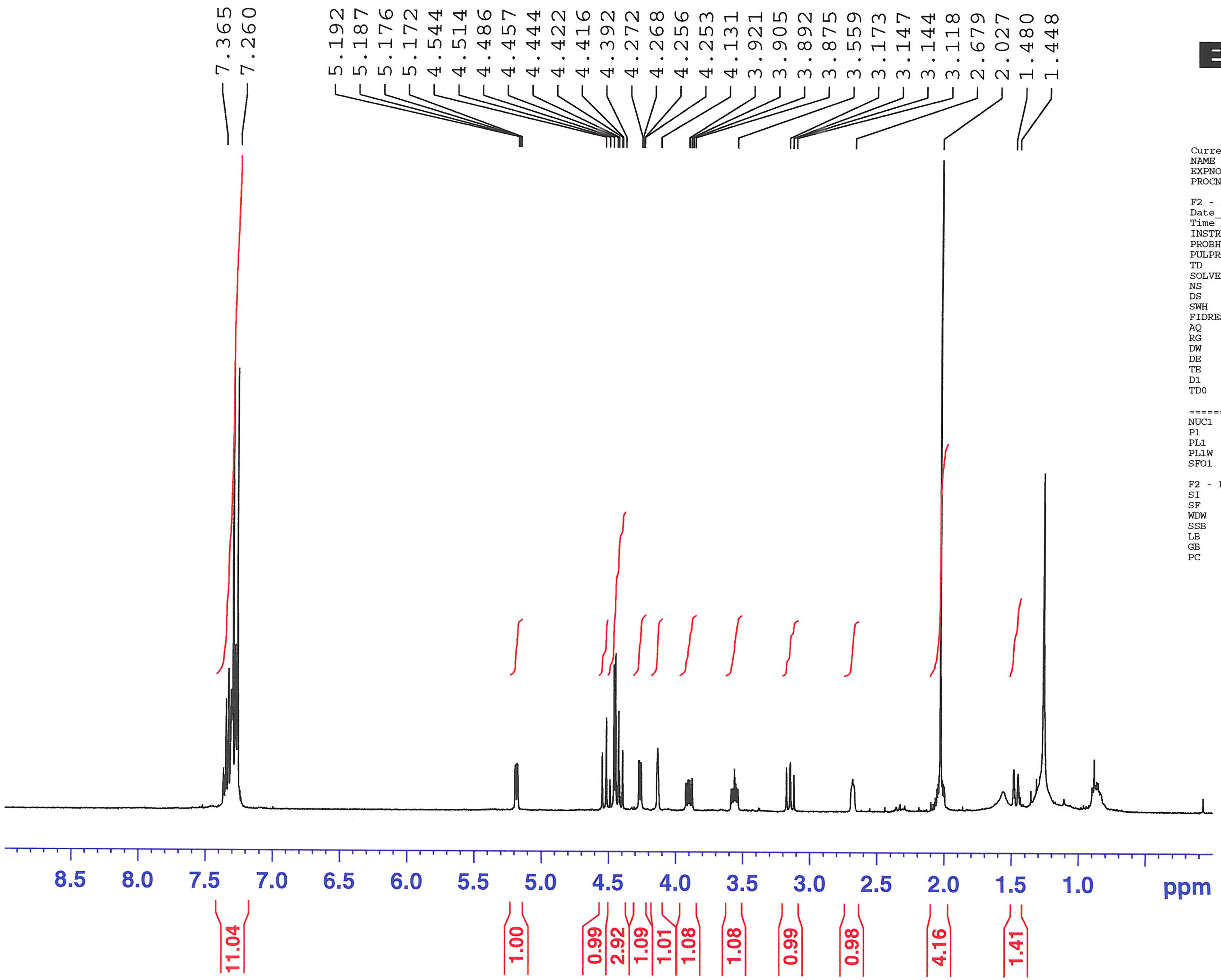

BRUKER

```
Current Data Parameters
NAME      JF-32-104-acetylation
EXPNO                10
PROCNO                1

F2 - Acquisition Parameters
Date_          20100423
Time               5.37
INSTRUM           spect
PROBHD   5 mm PABBO BB-
PULPROG            zg30
TD                65536
SOLVENT           CDCl3
NS                   16
DS                    2
SWH            8223.685 Hz
FIDRES         0.125483 Hz
AQ            3.9846387 sec
RG                  456
DW               60.800 usec
DE                 6.50 usec
TE                296.7 K
D1           1.00000000 sec
TD0                   1

======== CHANNEL f1 ========
NUC1                 1H
P1                 8.90 usec
PL1               -4.00 dB
PL1W        24.73352814 W
SFO1        400.1324710 MHz

F2 - Processing parameters
SI                32768
SF          400.1300096 MHz
WDW                  EM
SSB                   0
LB                 0.30 Hz
GB                    0
PC                 1.00
```

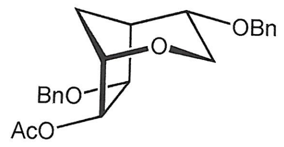

14

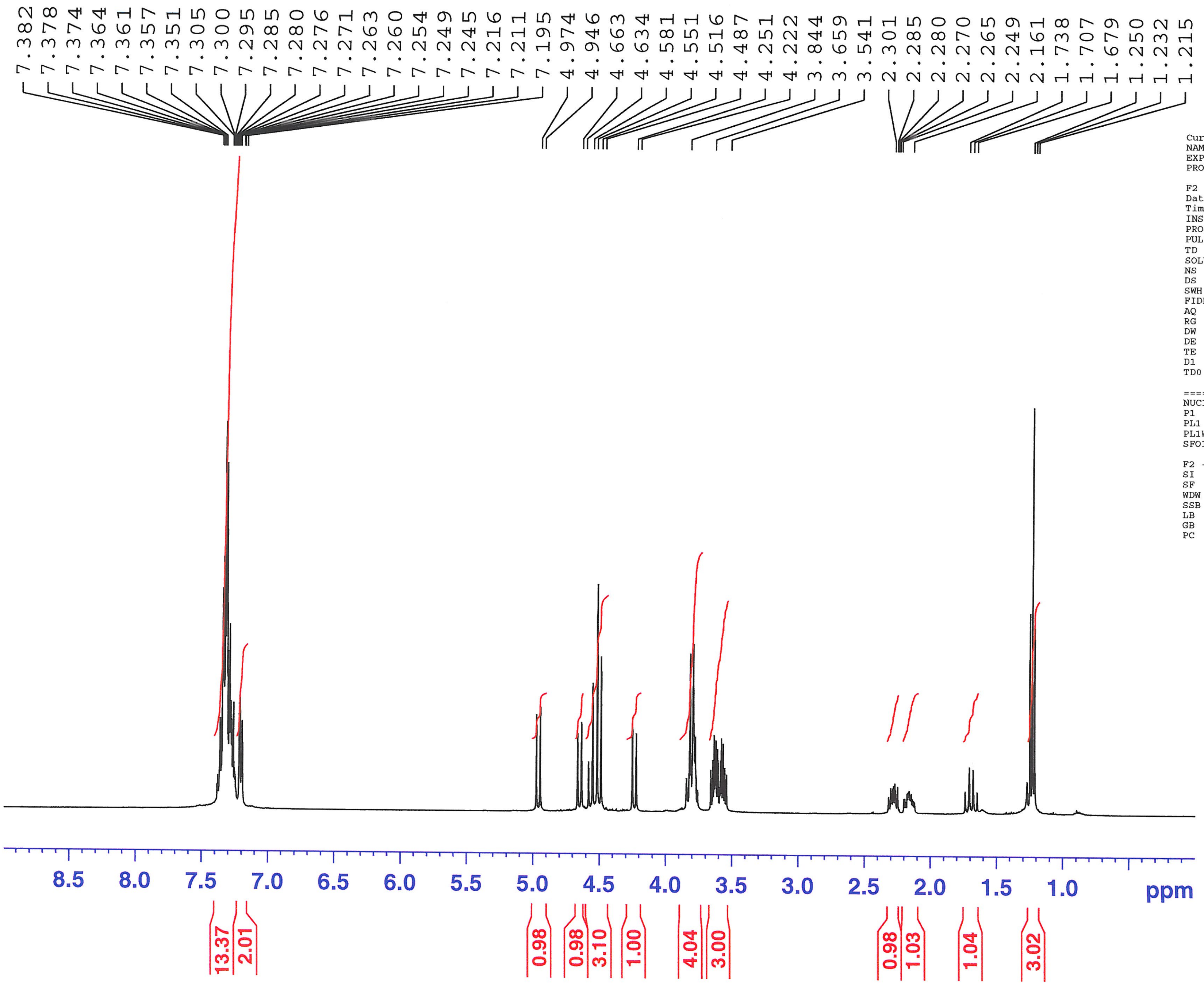


```
Current Data Parameters
NAME       JF-32-63-fr-6-13-swern
EXPNO                  10
PROCNO                  1

F2 - Acquisition Parameters
Date_            20091217
Time                 9.26
INSTRUM             spect
PROBHD    5 mm PABBO BB-
PULPROG              zg30
TD                  65536
SOLVENT             CDCl3
NS                     16
DS                      2
SWH              8223.685 Hz
FIDRES           0.125483 Hz
AQ              3.9846387 sec
RG                    203
DW                 60.800 usec
DE                   6.50 usec
TE                  297.4 K
D1             1.00000000 sec
TD0                     1

======== CHANNEL f1 ========
NUC1                   1H
P1                   8.90 usec
PL1                 -4.00 dB
PL1W          24.73352814 W
SFO1          400.1324710 MHz

F2 - Processing parameters
SI                  32768
SF            400.1300096 MHz
WDW                    EM
SSB                     0
LB                   0.30 Hz
GB                      0
PC                   1.00
```

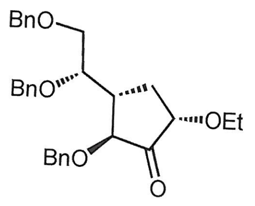

15

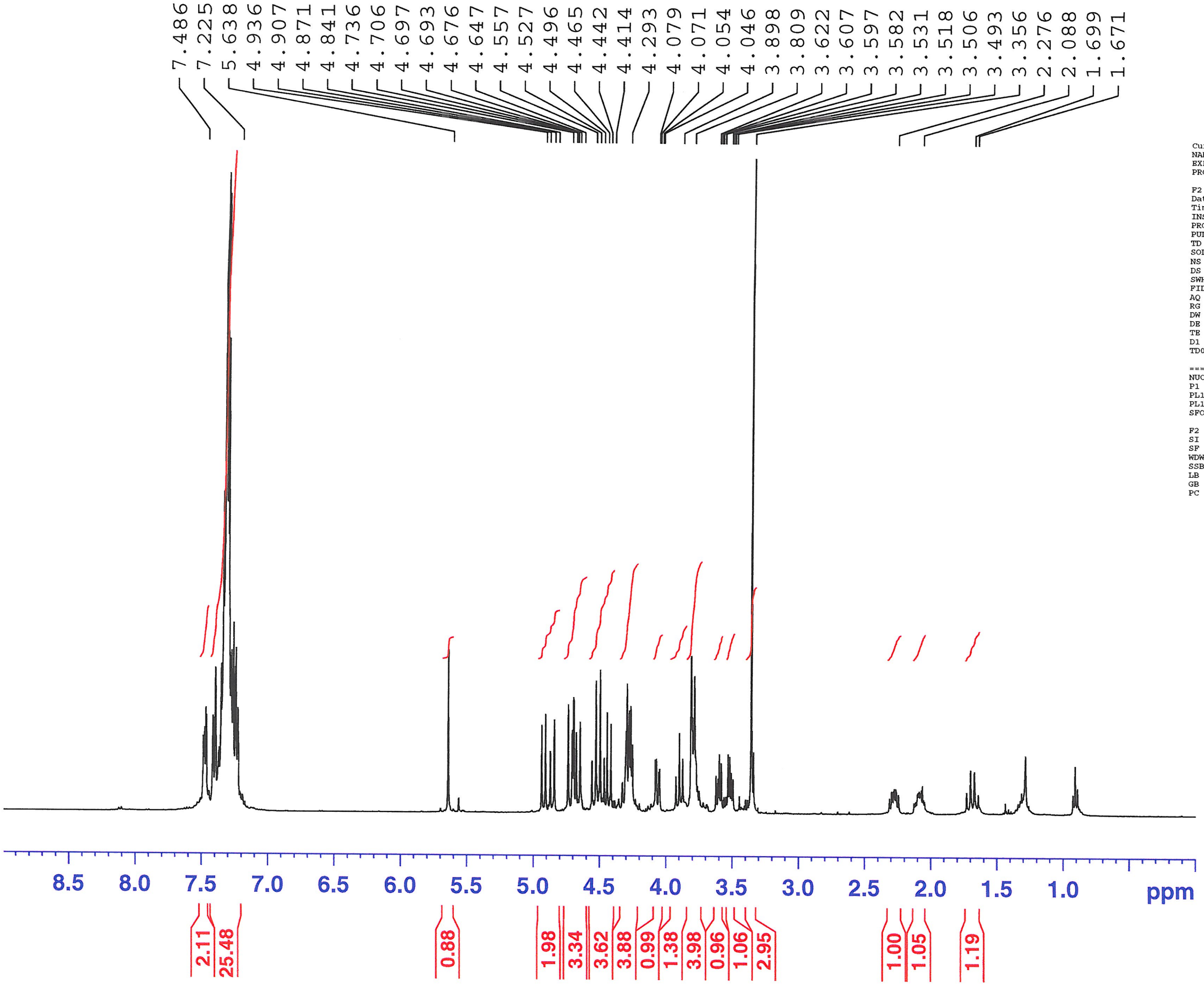

BRUKER

Current Data Parameters	
NAME	JP-32-91-fr-13-26-swernpseudo
EXPNO	10
PROCNO	1
F2 - Acquisition Parameters	
Date_	20100305
Time	10.57
INSTRUM	spect
PROBHD	5 mm PABBO BB-
PULPROG	zg30
TD	65536
SOLVENT	CDCl3
NS	16
DS	2
SWH	8223.685 Hz
FIDRES	0.125483 Hz
AQ	3.9846387 sec
RG	128
DW	60.800 usec
DE	6.50 usec
TE	297.0 K
D1	1.00000000 sec
TD0	1
======== CHANNEL f1 ========	
NUC1	1H
P1	8.90 usec
PL1	-4.00 dB
PL1W	24.73352814 W
SFO1	400.1324710 MHz
F2 - Processing parameters	
SI	32768
SF	400.1300093 MHz
WDW	EM
SSB	0
LB	0.30 Hz
GB	0
PC	1.00

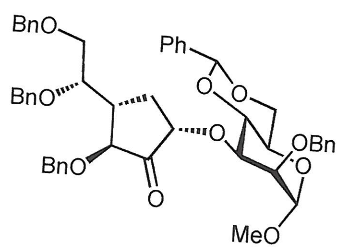

16

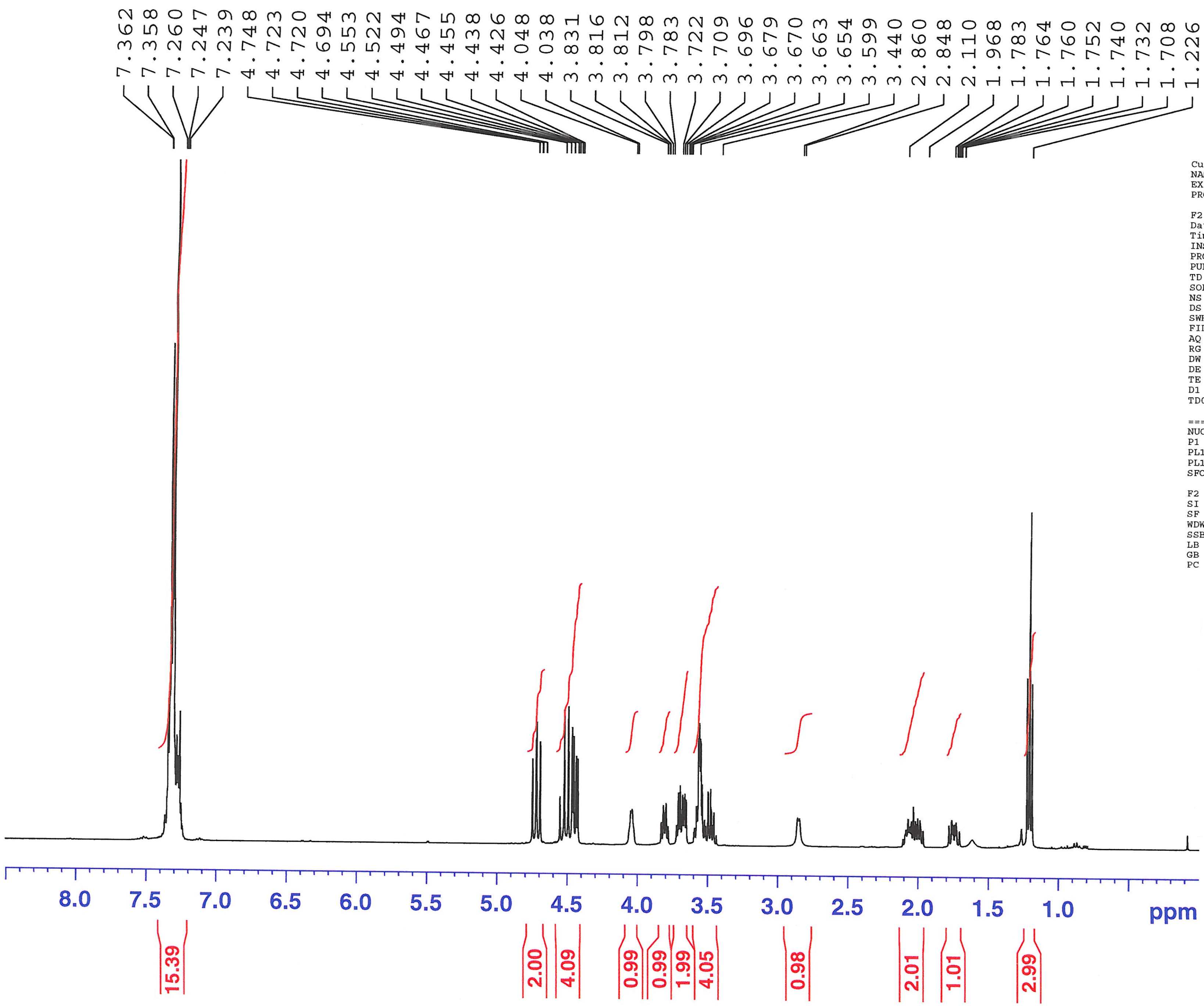

BRUKER

```
Current Data Parameters
NAME       JF-32-98-fr-11-15-galf
EXPNO                    10
PROCNO                    1

F2 - Acquisition Parameters
Date_              20100323
Time                   3.06
INSTRUM               spect
PROBHD    5 mm PABBO BB-
PULPROG                zg30
TD                    65536
SOLVENT               CDCl3
NS                       16
DS                        2
SWH                8223.685 Hz
FIDRES             0.125483 Hz
AQ                3.9846387 sec
RG                      228
DW                   60.800 usec
DE                     6.50 usec
TE                    296.7 K
D1               1.00000000 sec
TD0                       1

======== CHANNEL f1 ========
NUC1                     1H
P1                     8.90 usec
PL1                   -4.00 dB
PL1W            24.73352814 W
SFO1            400.1324710 MHz

F2 - Processing parameters
SI                    32768
SF              400.1300097 MHz
WDW                      EM
SSB                       0
LB                     0.60 Hz
GB                        0
PC                     1.00
```

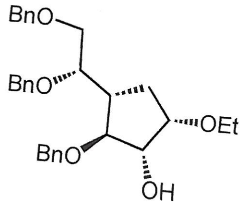

17

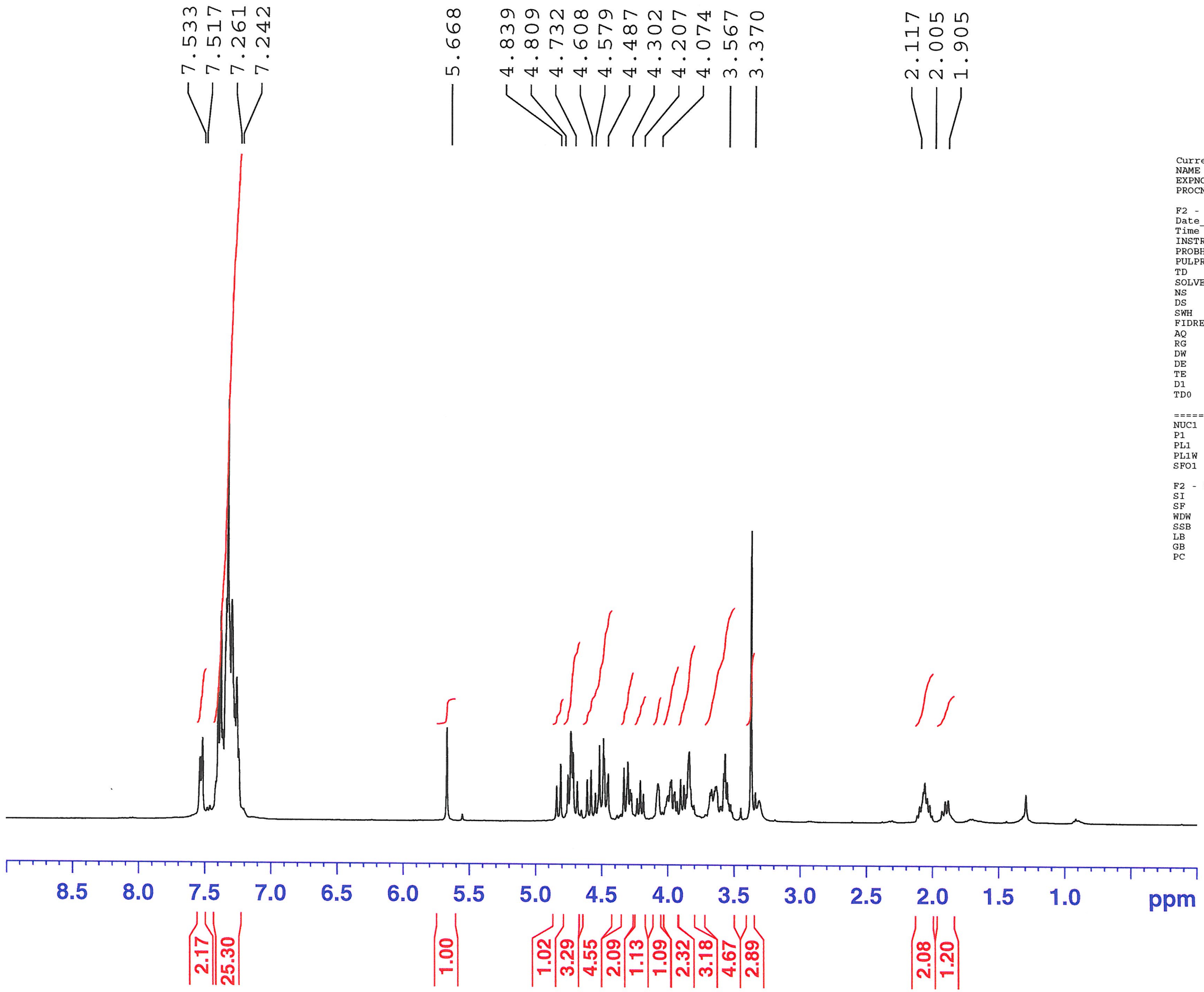

Current Data Parameters

NAME	JF-32-106-FL2-fr-33-49
EXPNO	10
PROCNO	1

F2 - Acquisition Parameters

Date_	20100505
Time	6.50
INSTRUM	spect
PROBHD	5 mm PABBO BB-
PULPROG	zg30
TD	65536
SOLVENT	CDCl3
NS	16
DS	2
SWH	8223.685 Hz
FIDRES	0.125483 Hz
AQ	3.9846387 sec
RG	101
DW	60.800 usec
DE	6.50 usec
TE	297.0 K
D1	1.00000000 sec
TD0	1

======== CHANNEL f1 ========

NUC1	1H
P1	8.90 usec
PL1	-4.00 dB
PL1W	24.73352814 W
SFO1	400.1324710 MHz

F2 - Processing parameters

SI	32768
SF	400.1300116 MHz
WDW	EM
SSB	0
LB	0.30 Hz
GB	0
PC	1.00

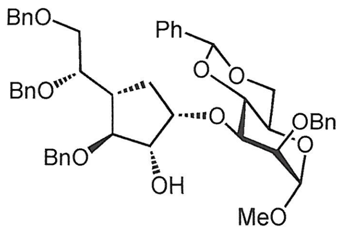

18
